# Unraveling the Protective Role of Oleocanthal and Its Oxidation Product, Oleocanthalic Acid, against Neuroinflammation

**DOI:** 10.3390/antiox13091074

**Published:** 2024-09-03

**Authors:** Maria Cristina Barbalace, Michela Freschi, Irene Rinaldi, Lorenzo Zallocco, Marco Malaguti, Clementina Manera, Gabriella Ortore, Mariachiara Zuccarini, Maurizio Ronci, Doretta Cuffaro, Marco Macchia, Silvana Hrelia, Laura Giusti, Maria Digiacomo, Cristina Angeloni

**Affiliations:** 1 Department for Life Quality Studies, Alma Mater Studiorum, University of Bologna, Corso d’Augusto 237, 47921 Rimini, Italy; maria.barbalace2@unibo.it (M.C.B.); michela.freschi@irst.emr.it (M.F.); irene.rinaldi5@unibo.it (I.R.); marco.malaguti@unibo.it (M.M.); silvana.hrelia@unibo.it (S.H.); cristina.angeloni@unibo.it (C.A.); 2 Biostatistics and Clinical Trials Unit, IRCCS Istituto Romagnolo per lo Studio dei Tumori (IRST) “Dino Amadori”, 47014 Meldola, Italy; 3 Department of Translational Research and New Technologies in Medicine and Surgery, University of Pisa, 56126 Pisa, Italy; l.zallocco@gmail.com; 4 Department of Pharmacy, University of Pisa, 56126 Pisa, Italy; clementina.manera@unipi.it (C.M.); gabriella.ortore@unipi.it (G.O.); doretta.cuffaro@unipi.it (D.C.); marco.macchia@unipi.it (M.M.); maria.digiacomo@unipi.it (M.D.); 5 Department of Medical, Oral and Biotechnological Sciences, University G. D’Annunzio of Chieti-Pescara, 66100 Chieti, Italy; mariachiara.zuccarini@unich.it (M.Z.); maurizio.ronci@unich.it (M.R.); 6 COIIM—Interuniversitary Consortium for Engineering and Medicine, 86100 Campobasso, Italy; 7 Interdepartmental Research Center “Nutraceuticals and Food for Health”, University of Pisa, 56100 Pisa, Italy; 8 School of Pharmacy, University of Camerino, 62032 Camerino, Italy

**Keywords:** oleocanthal, oleocanthalic acid, neuroinflammation, BV-2 microglial cells, lipopolysaccharide, TLR4, ACOD1, gelsolin, 14-3-3 protein family, clathrin

## Abstract

Neuroinflammation is a critical aspect of various neurodegenerative diseases, such as Alzheimer’s and Parkinson’s diseases. This study investigates the anti-neuroinflammatory properties of oleocanthal and its oxidation product, oleocanthalic acid, using the BV-2 cell line activated with lipopolysaccharide. Our findings revealed that oleocanthal significantly inhibited the production of pro-inflammatory cytokines and reduced the expression of inflammatory genes, counteracted oxidative stress induced by lipopolysaccharide, and increased cell phagocytic activity. Conversely, oleocanthalic acid was not able to counteract lipopolysaccharide-induced activation. The docking analysis revealed a plausible interaction of oleocanthal, with both CD14 and MD-2 leading to a potential interference with TLR4 signaling. Since our data show that oleocanthal only partially reduces the lipopolysaccharide-induced activation of NF-kB, its action as a TLR4 antagonist alone cannot explain its remarkable effect against neuroinflammation. Proteomic analysis revealed that oleocanthal counteracts the LPS modulation of 31 proteins, including significant targets such as gelsolin, clathrin, ACOD1, and four different isoforms of 14-3-3 protein, indicating new potential molecular targets of the compound. In conclusion, oleocanthal, but not oleocanthalic acid, mitigates neuroinflammation through multiple mechanisms, highlighting a pleiotropic action that is particularly important in the context of neurodegeneration.

## 1. Introduction

Neurodegenerative diseases, such as Alzheimer’s disease (AD) and Parkinson’s disease (PD), are highly devastating disorders affecting millions of people worldwide. They are characterized by the progressive loss of neurons in the central or peripheral nervous system, leading to motor, sensory, and/or cognitive defects [1]. Unfortunately, for most of them, no effective disease-modifying pharmacological therapy exists [2,3]. Although these diseases exhibit distinct pathogenetic mechanisms, including diverse protein aggregates and genetic variations, they all share common characteristics like oxidative stress and persistent neuroinflammation [4,5]. Neuroinflammation, or, more specifically, the activation of the neuroimmune cells into pro-inflammatory states, is an effective endogenous defense that protects the central nervous system (CNS) against microorganisms and injuries [6]. Nevertheless, persistent neuroinflammatory processes may culminate in a cascade of events, ultimately allowing for the development of a chronic inflammation state associated with progressive neuronal damage [7]. Among the neuroimmune cells, microglia play a pivotal role in neuroinflammation. Activated microglia can polarize into either M1 pro-inflammatory or M2 anti-inflammatory phenotypes in response to different micro-environmental disturbances with significant implications in neurodegenerative diseases [8,9,10]. This is not a rigid classification; instead, microglia can switch from one phenotype to another, exhibiting a continuum of intermediate phenotypes between M1 and M2 [10,11]. M1 microglia detect harmful stimuli through various receptors like nucleotide-binding oligomerization domains (NODs), NOD-like receptors, toll-like receptors (TLRs), and multiple scavenger receptors and release pro-inflammatory factors, leading to inflammation and neuronal death [12]. Among the stimuli that activate microglial cells, the most extensively studied is lipopolysaccharide (LPS). LPS binds to TLR4, inducing a series of intracellular signaling cascades that lead to the production of reactive oxygen species (ROS), nitric oxide (NO), and inflammatory cytokines [13,14]. Conversely, M2 microglia, activated by interleukin (IL)-4, IL-10, or IL-13, release anti-inflammatory cytokines and promote repair and regeneration, exerting neuroprotective effects. The delicate balance between M1 and M2 activation is crucial for maintaining brain homeostasis and preventing neurodegenerative diseases [10]. Therefore, identifying compounds that can positively modulate neuroinflammation represents a potential strategy for addressing neurodegenerative diseases. This is further supported by studies conducted both in vitro and in vivo, which suggest that exposure to nonsteroidal anti-inflammatory drugs (NSAIDs) is associated with a reduced risk of AD [15] and PD [16]. Since the use of anti-inflammatory medications for the primary prevention of neurodegenerative diseases cannot be considered a viable option due to both costs and potential side effects, the identification of compounds present in the typical diet with anti-inflammatory properties could represent a suitable solution for this purpose. In addition, numerous studies support that plant bioactive metabolites exert pleiotropic effects [17], modulating multiple metabolic pathways through a variety of molecular targets, probably due to their molecular promiscuity and character diversity acquired through evolution [18]. Due to the multifactorial nature of neurodegenerative diseases, the pleiotropic activity of natural compounds represents an added value in counteracting these pathologies.

Increasing studies are showing a link between the Mediterranean diet and a lower risk of cognitive impairment [19,20], improved cognitive function, and a lower incidence of neurodegenerative diseases [19,21], including Alzheimer’s disease [22]. A distinctive feature of the Mediterranean diet is the use of extra virgin olive oil (EVOO) as the main source of fat (30 to 50 g/day) [23,24]. In particular, EVOO has been widely associated with neuroprotective properties, mainly attributed to its phenolic compounds, which exert antioxidant and anti-inflammatory activities [23,25]. Two main fractions can be distinguished in the chemical composition of fresh EVOO: the saponifiable fraction (about 98–99%) consisting mainly of monounsaturated and polyunsaturated fatty acids (MUFA and PUFA) and the unsaponifiable fraction (only 2%) [26,27,28]. That latter fraction consists of more than 230 compounds, among which polyphenols, such as phenolic alcohols, flavonoids, secoiridoids, lignans, phenyl-acids, and hydroxy-isochromanes [26,28,29,30], stand out for their biological properties. Secoiridoids constitute more than 90% of these compounds [31]. This class of compounds is mainly represented by oleacein (OLa) and oleocanthal (OL), which are produced through successive action of β-glucosidase and methylesterase on the glycosidic forms of oleuropein and ligstroside, respectively [27]. OL and OLa constitute the most important portion of fresh EVOO [26,29] due to their high concentration and several biological effects [30,32,33]. Between the two, OL, which is mainly responsible for EVOO’s unique pungent taste and burning sensation in the throat [34,35], has aroused the most interest in the scientific community. The great appeal of this compound was due to the well-documented strong anti-inflammatory properties of dose-dependent cyclooxygenase (COX)-1 and COX-2 inhibition with an intensity comparable to the well-known nonsteroidal anti-inflammatory (NSAID) ibuprofen [34]. For this reason, OL is often referred to as a naturally occurring NSAID [36,37]. Several studies have investigated the neuroprotective effect of OL [30,38,39,40,41]. However, none of these studies have focused on its potential action against neuroinflammation. Exclusively, OL anti-inflammatory activity has been observed in human primary osteoarthritis chondrocytes [36] and in murine peritoneal macrophages activated with LPS [42].

The nutrional quality of EVOO is strongly influenced by the content of OL and other phenolic compounds. During storage, EVOO normally undergoes degradation processes, such as hydrolytic and auto-oxidation processes, which inevitably change its phenolic composition over time [26,43,44]. The main hydrolytic product of OL is tyrosol, while oleocanthalic acid (OA) represents a newly discovered auto-oxidation derivative [30,45].

To date, the biological activities of OA have been relatively underexplored. A couple of studies demonstrated its antioxidant activity in vitro [30,45], while another study, primarily focused on the chemical characteristics of OA, showed that it increased the expression of P-gp, LRP1, ZO1, and claudin-5 in the immortalized mouse brain endothelial cell line, bEnd3 [43]. On these premises, the aim of this study was to investigate the potential anti-inflammatory activity of OL and its metabolite OA using the microglial cell line BV-2 activated with LPS as an in vitro model of neuroinflammation.

## 2. Materials and Methods

### 2.1. Chemicals

OC and OA were isolated as pure standards from EVOO using the methodology outlined in our previous studies [30]. Dulbecco’s modified Eagle’s medium (DMEM), penicillin/streptomycin, L-glutamine solution, phosphate-buffered saline (PBS), 3-(4,5-dimethylthiazol-2-yl)-2,5-diphenyltetrazolium bromide (MTT), 2′,7′-dichlorodihydrofluorescein diacetate (DCFH-DA), monochlorobimane (MCB), LPS from Escherichia coli serotype O127:B8, primers for real-time polymerase chain reaction (RT-PCR), RIPA buffer, sodium pyrophosphate, phenylmethylsulfonyl fluoride, FITC-dextran 40 kDa, paraformaldehyde, dimethyl sulfoxide (DMSO), Griess reagent, sodium nitrite (NaNO_2_), and all other chemicals of the highest analytical grade were purchased from Merck Italia (Milan, Italy). Low-endotoxin fetal bovine serum (low-endotoxin FBS) was purchased from Euroclone (Milan, Italy). RNeasy Mini Kit was from Qiagen (Hilden, Germany). iScript™ cDNA Synthesis Kit was purchased from Bio-Rad (Hercules, CA, USA). Excel-Taq FAST qPCR SybrGreen (no ROX) was purchased from SMOBIO Technology, Inc (Hsinchu City, Taiwan).

### 2.2. Cell Culture and Treatments

BV-2 murine microglial cells were kindly provided by Prof. Elisabetta Blasi (University of Modena and Reggio Emilia, Italy). BV-2 cells were cultured in DMEM supplemented with 10% (*v*/*v*) low-endotoxin FBS, 2 mM L-glutamine, 50 U/mL penicillin, and 50 μg/mL streptomycin, and they were maintained in a humidified incubator with 5% CO_2_ at 37 °C as reported in [39]. Cells were pre-treated for 2 h with OL and OA (10 μM unless otherwise specified, from a stock solution of 10 mM in DMSO) before the addition of LPS at a final concentration of 100 ng/mL for an additional 1 h or 24 h. Control cells were treated with vehicle (DMSO at the same dilution as the investigated compounds). At the end of the treatments, cell morphology images were taken using the microscope Eclipse Ti-E (Nikon Instruments Spa, Florence, Italy) DS-U3 camera and NIS-Elements BR 3.2 64-bit Software.

### 2.3. Cell Viability Assay

MTT assay was used to evaluate cell viability as previously reported [39]. BV-2 microglial cells were seeded at the density of 1.0 × 10^4^ cell/well into 96-well plates. For the assessment of the cytotoxicity of OL and OA, cells were exposed for 24 h to different concentrations (1, 10, 50, and 100 μM) of the two compounds, while for the analysis of the protective effect of these molecules from the LPS-induced cytotoxicity, BV-2 was pre-treated for 2 h with 10 μM of OL and OA, and then LPS was added at a final concentration of 100 ng/mL for 24 h. At the end of each experiment, cells were incubated with MTT solution (0.5 mg/mL) for 30 min at 37 °C, and then the MTT solution was replaced with 200 μL of DMSO to dissolve the formed formazan crystals. Absorbance at 595 nm was measured using a multilabel plate spectrophotometer (VICTOR3 V Multilabel Counter; Perkin–Elmer, Wellesley, MA, USA). Cell viability is expressed as % of control.

### 2.4. NO Production

Microglial NO production was evaluated by measuring the levels of nitrite released into the culture media, using the Griess reagent, as previously reported [39]. BV-2 cells were seeded in 24-well plates at the density of 4.2 × 10^4^ cell/well, pre-treated for 2 h with different concentrations of OL and OA (0.1, 1, 5, and 10 μM), and then LPS was added at a final concentration of 100 ng/mL for another 24 h. Subsequently, cell media were collected and centrifuged at 300× *g* for 5 min at 4 °C to remove any residual cells or cell debris. Fifty microliters of supernatant from each sample were mixed with an equal volume of Griess reagent in a 96-well assay plate. After incubating the plate for 15 min, the absorbance was measured at 540 nm using a multilabel plate spectrophotometer (VICTOR3 V Multilabel Counter; Perkin–Elmer, Wellesley, MA, USA). Nitrite concentration in the samples was determined by reference to a standard curve generated with known concentrations of NaNO_2_.

### 2.5. DCFH-DA Assay

The intracellular ROS level in BV-2 microglial cells was assessed using the DCFH-DA fluorescent probe. Cells were seeded at the density of 1.0 × 10^4^ cell/well into 96-well plates and treated as described previously. Subsequently, BV-2 cells were exposed to a solution of 10 μM DCFH-DA (diluted in DMEM without fetal bovine serum and phenol red) for 30 min at 37 °C in the dark. After this incubation period, the probe was replaced with PBS, and cell fluorescence was quantified using a multilabel plate reader (VICTOR3 V Multilabel Counter; Perkin–Elmer, Wellesley, MA, USA) at excitation and emission wavelengths of 485 nm and 535 nm, respectively. Intracellular ROS levels were expressed as a percentage of control cells.

### 2.6. MCB Assay

The intracellular levels of reduced GSH in BV-2 microglial cells were determined using the MCB fluorometric assay. Cells were seeded at the density of 1.0 × 10^4^ cell/well into 96-well plates and treated as described previously. Subsequently, BV-2 cells were exposed to a solution of 50 μM of MCB (diluted in DMEM without fetal bovine serum and phenol red) for 30 min at 37 °C in the dark. After this incubation period, the probe was replaced with PBS, and cell fluorescence was quantified using a multilabel plate reader (VICTOR3 V Multilabel Counter; Perkin–Elmer, Wellesley, MA, USA) at excitation and emission wavelengths of 355 nm and 460 nm, respectively. GSH levels were expressed as a percentage of control cells.

### 2.7. Real-Time Polymerase Chain Reaction (PCR)

BV-2 microglial cells were treated as described above, and then total RNA was extracted from each sample using the RNeasy Mini Kit (Qiagen, GmbH, Hilden, Germany) following the manufacturer’s instructions. The yield and purity of the extracted RNA were evaluated using the NanoVue spectrophotometer (GE Healthcare, Milan, Italy). Subsequently, cDNA was generated by reverse-transcribing 1 μg of total RNA using the iScript cDNA Synthesis Kit (BIO-RAD, Hercules, CA, USA). PCR was then conducted in a total volume of 10 μL, comprising 5 μL Excel-Taq FAST qPCR SybrGreen (SMOBIO Technology, Inc. Hsinchu City, Taiwan), 2.5 μL (12.5 ng) of cDNA, and 0.4 μL (400 nM of final concentration) of each primer, as reported in [39]. The primers used are listed in Table 1. cDNA amplification was performed using the CFX Connect^TM^ thermal cycler (BIO-RAD, Hercules, CA, USA), starting with polymerase activation at 95 °C for 30 s, followed by 40 cycles of 5 s at 95 °C and 30 s at 60 °C. To ensure quality control and generation of a single product, melt curves were run. GAPDH was used as a reference gene. Normalized expression levels were calculated by comparing them to control cells via the 2^−ΔΔCT^ method.

### 2.8. Preparation of Nuclear and Cytosolic Fractions

The nuclear and cytoplasmic fractions were isolated from BV-2 cells and prepared according to the manufacturer’s instructions for the Nuclear Extract kit (Active Motif, Waterloo, Belgium). All steps were performed at 4 °C or on ice unless stated otherwise.

### 2.9. Western Immunoblotting

Cells were treated as described above, then rinsed with ice-cold PBS, followed by cell lysis using an RIPA buffer solution containing mammalian Protease Inhibitor Cocktail (1:100 dilution), 10 mg/mL of phenylmethylsulfonyl fluoride, and PhosSTOP 1X (Roche, Mannheim, Germany) for the total protein content. Subsequently, all cell lysates were boiled for 5 min at 96 °C and separated by electrophoresis on SDS-polyacrylamide gels (4–20%) (BIO-RAD, Hercules, CA, USA). Protein transfer to a nitrocellulose membrane (0.45 μm, BIO-RAD, Hercules, CA, USA) was conducted in Tris-glycine-methanol buffer at 110 V for 90 min. The membranes were blocked using EveryBlot Blocking Buffer (BIO-RAD, Hercules, CA, USA), then incubated overnight at 4 °C on a 3D rocking table with primary antibodies against iNOS (#13120), COX-2 (#12282), NLRP3 (#15101), NF-κB (#8242), p- NF-κB (#3031), p38(#9212), p-p38 (#9211), ERK 1/2 (#9102), p-ERK 1/2 (#9101), Akt (#9272), p-Akt (#9271) (cell-signaling technology, Beverly, MA) (1:1000 dilution) and anti-β-actin (A5441, Sigma–Aldrich–Merck, Saint Louis, MO 63103, USA) (1:5000 dilution) as internal loading control for cytoplasmic extracts, and Lamin A/C (#2032, cell-signaling technology, Beverly, MA) (1:1000 dilution). After the incubation with the corresponding secondary antibodies, the membranes were exposed to Clarity™ Western ECL Substrate (BIO-RAD, Hercules, CA, USA) to visualize the marked proteins. ImageLab software version 5.2 was used to conduct the densitometric analysis of specific immunolabeled bands.

### 2.10. TLR4 Surface Expression

BV-2 cells were seeded in T25 flasks (1.4 × 10^6^ cells/flask) and treated as previously described. At the end of each experiment, cells were washed and detached with ice-cold PBS, then centrifuged at 300× *g* for 5 min. The resulting cell pellet was washed with PBS, centrifuged at 300× *g* for 5 min, and then fixed with paraformaldehyde 2%. Fixed cells were washed twice with washing buffer (PBS + 1% BSA). After removing the supernatant, cells were resuspended with FITC-conjugated rabbit anti-TLR4 antibody (Stressmarq, cat. no. SPC-200) and 1:100 dilution in PBS + 1% BSA, then incubated for 30 min in the dark at 37 °C. After antibody incubation, cells were washed twice as described above, then resuspended with PBS at the appropriate dilution for flow cytometer analysis/running. Samples were analyzed using the Attune™ NxT Acoustic Focusing Cytometer (Thermo Fisher Scientific, Waltham, MA, USA).

### 2.11. CD14 Surface Expression

BV-2 cells were seeded in T25 flasks (1.4 × 10^6^ cell/flask) and treated as previously described. At the end of each experiment, cells were washed and detached with ice-cold PBS, then centrifuged at 300× *g* for 5 min. The resulting cell pellet was then washed with PBS, centrifuged at 300× *g* for 5 min, and resuspended with PE-conjugated mouse anti-CD14 antibody (Miltenyi Biotec, cat. no. 130-115-558, 51429 Bergisch Gladbach, Germany), 1:50 dilution in PBS + 1% BSA, then incubated for 45 min in the dark at 4 °C. After antibody incubation, cells were washed twice with washing buffer (PBS + 1% BSA) and fixed with paraformaldehyde 2% for 10 min. Fixed cells were washed twice, as described above. After removing the supernatant, cells were resuspended with PBS at the appropriate dilution for flow cytometer analysis/running. Samples were analyzed using the Attune™ NxT Acoustic Focusing Cytometer (Thermo Fisher Scientific).

### 2.12. BV-2 Phagocytic Activity

BV-2 cells were seeded in T25 flasks (1.4 × 10^6^ cells/flask) and treated as previously described. Then, cells were washed with DMEM 1% FBS without phenol red and incubated with FITC-dextran 40 kDa (Sigma–Aldrich–Merck, Saint Louis, MO 63103, USA) at the final concentration of 1 mg/mL (diluted in DMEM 1% FBS withoutphenol red) for 1 h in the dark at 37 °C (on ice for the negative control, NC). After the incubation, cells were washed and detached with ice-cold PBS, then centrifuged at 300× *g* for 5 min. The resulting cell pellet was extensively washed with DMEM 1% FBS without phenol red and then appropriately diluted for flow cytometer analysis/running. Samples were analyzed using the Attune™ NxT Acoustic Focusing Cytometer (Thermo Fisher Scientific).

### 2.13. Molecular Docking Studies

OL and OA were built and optimized using Maestro [46]. Crystallographic structures 5IJB [47] and 1WWL [48], relative to ligand-free mTLR4-MD2 and mCD14, respectively, already refined through Maestro, were used for docking OL and OA using the GOLD program [49]. For the blind docking in the ligand-free mTLR4-MD2 (5IJB) structure, the region of interest was defined using the ligand coordinates of the superposed mTLR4-MD2-lipidA complex [47], such that the 5IJB protein contained all residues within 30 Å of the experimental ligand. The blind docking in CD14 was performed using a radius of 30 Å from the three NAG molecules contained in the PDB structure 1WWL. The “allow early termination” command was always deactivated. All ligands were submitted to 40 genetic algorithm runs for docking, using the scoring functions Chemscore, ASP, and PLP available in GOLD. The output orientations were clustered based on an RMSD distance of 2.0 Å, and the three most frequent poses within the top five clusters of all scoring functions were selected. The default GOLD parameters were used for all other variables. Docking results were analyzed by using Chimera 1.16 [50].

### 2.14. Proteomic Analysis

For proteomic analysis, BV-2 cells were treated for 2 h with OL before the addition or not of LPS for further 24 h, as described above. At the end of the treatments, cells were collected and washed with PBS. After centrifugation (1000× *g* for 5 min), the resulting pellets were immediately frozen and stored at −80 °C until use. 2DE was conducted as previously described [51]. Cellular protein extracts (200 µg of proteins) were separated on IPG blue strips (Serva, Heidelberg, Germany) of 18 cm, with a linear gradient pH 3–10 for the first dimension, followed by 12% SDS-PAGE for the second dimension. The gels were stained with Ruthenium II tris (bathophenanthroline disulfonate) tetrasodium salt (Cyanagen, Bologna, Italy) (RuBP). ImageQuant LAS4010 (GE Health Care, CA, USA) was used to acquire images. The analysis of images was performed using Same Spot (v4.1, TotalLab, Newcastle Upon Tyne, UK) software. The spot volume ratios between the different conditions were calculated using the average spot normalized volume of the three biological replicates. The software calculated the significance of the differences in normalized volume for each spot including the analysis of variance (ANOVA test). The protein spots that showed fold expression ≥ 1.5, *p*-value < 0.05, and *q*-value < 0.05 were identified by mass spectrometry.

### 2.15. Spot Digestion and Protein Identification

For protein identification, selected spots were cut out from gel, and trypsin was digested as described [51]. Tryptic peptides were analyzed by LC-MS/MS using an UltiMate3000 RSLCnano chromatographic system coupled to an Orbitrap Fusion Tribrid mass spectrometer (Thermo Fisher Scientific, Waltham, MA, USA), operating in positive ionization mode, equipped with a nanoESI source (EASY-Spray NG) [52].

Raw data were directly loaded in PEAKS Studio Xpro software (v10.6 build 2020122, Bioinformatic Solutions Inc, Waterloo, ON, Canada) using the “correct precursor only” option. The mass lists were searched against the Uniprot/SwissProt database (downloaded June 2021) to which a list of common contaminants was appended, selecting Mus musculus taxonomy (566155 searched entries). Non-specific cleavage was allowed to one end of the peptides, with a maximum of two missed cleavages and two variable PTMs per peptide. Then, 10 ppm and 0.5 Da were set as the highest error mass tolerances for precursors and fragments, respectively. Also, −10lgP threshold for PSMs was manually set to 35.

### 2.16. Bioinformatic Analysis

Enrichment analysis was performed using Metascape [53] to obtain GO biological processes and QIAGEN’s Ingenuity Pathway Analysis (IPA, QIAGEN Redwood City, USA, www.qiagen.com/ingenuity, Content version: 21249400, accessed 24 August 2023) to determine the predominant canonical pathways whose activity appears to change in a significant manner according to the activation z-score, *p*-value, and ratio. The ratio is meant as an indicator of which pathway has been affected the most based on the bulk overlap of genes uploaded into IP.

The graphs were prepared with SRplot [54]. Graph Pad prism (Prism 7; GraphPad Software, San Diego, CA, USA) was used to represent the Volcano plot.

### 2.17. Statistical Analysis

Each experiment was performed at least three times, and all values are represented as mean ± SEM. One-way analysis of variance (ANOVA) was used to compare differences among groups, followed by Dunnett’s test or Turkey’s multiple comparisons test (GraphPad Prism 9.4.1, San Diego, CA, USA). Values of *p* < 0.05 were considered statistically significant.

## 3. Results

### 3.1. Evaluation of the Oleocanthal and Oleocanthalic Acid Potential Cytotoxicity

**Figure 1:**
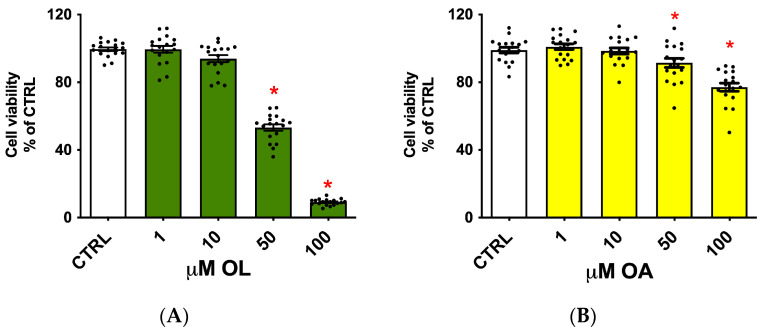
Viability of BV-2 cells treated with different concentrations of OL and OA. BV-2 cells were exposed to increasing concentrations of OL (**A**) and OA (**B**) (1–10–50–100 µM) for 24 h, and cell viability was evaluated by MTT assay. Data are represented as % of CTRL. Each bar represents means ± SEM of three independent experiments (six units per group). Data were analyzed using one-way ANOVA followed by Dunnett’s test. * *p* < 0.05 vs. CTRL.

To evaluate OL and OA potential cytotoxicity, cells were exposed to increasing concentrations of OL and OA (1–10–50–100 µM) for 24 h, and the cell viability was assessed by MTT assay (Figure 1). Both compounds showed cytotoxicity at the highest concentrations (50 and 100 µM). OL strongly and significantly reduced cell viability to around 50% and 10%, respectively (A). OA demonstrated a similar trend but with a minor impact on cell viability (B). Based on these results, we decided to treat cells with concentrations up to 10 µM in the following experiments.

### 3.2. Effect of OL and OA Treatment on NO Production in LPS-Activated BV-2 Cells

NO levels in the culture medium were used as a marker of the BV-2 cell activation. Cells were treated with increasing concentrations of OL and OA (0.1–1–5–10 µM) for 2 h, and then exposed to 100 ng/mL LPS for 24 h. At the end of the experiment, NO release in the culture medium was quantified by the Griess reagent.

**Figure 2:**
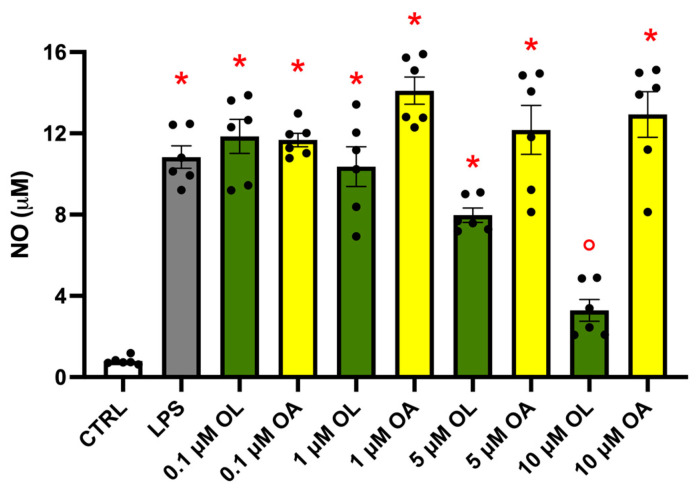
NO levels in BV-2 cells exposed to different concentrations of OL and OA. BV-2 cells were pre-treated for 2 h with increasing concentrations of OL and OA (0.1–1–5–10 µM) and then co-treated with 100 ng/mL LPS for 24 h. NO release was quantified by the Griess reagent. Data are represented as µM of NO released in the culture medium. Each bar represents means ± SEM of at least three independent experiments (two units per group). Data were analyzed by one-way ANOVA followed by Tukey’s test. * *p* < 0.05 vs. CTRL; ° *p* < 0.05 vs. LPS.

As foreseeable, LPS treatment induced a strong release of NO with respect to control cells (Figure 2). Then, 5 µM of OL significantly reduced NO levels compared to LPS-treated cells, while 10 µM of OL not only significantly reduced NO levels compared to LPS but even maintained NO levels at a value comparable to those observed in control cells. This suggests that 10 µM of OL possesses potent inhibitory effects on NO production. On the contrary, OA did not modify NO levels with respect to LPS at any tested concentrations. With these results, we decided to use 10 µM of OL and OA in the subsequent experiments.

### 3.3. OL Counteracts LPS-Induced Damage in BV-2 Cells

**Figure 3:**
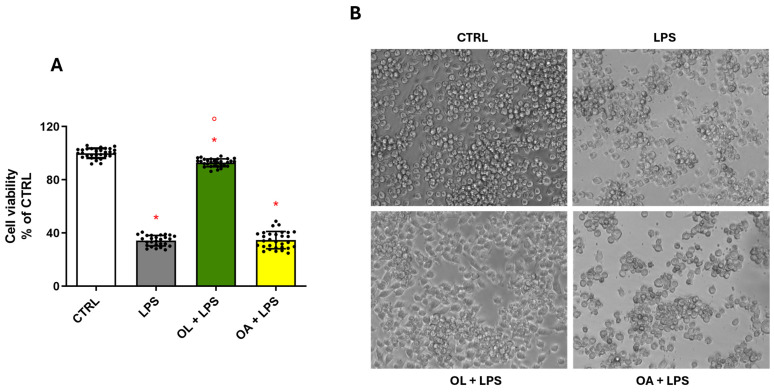
Cytoprotective effect of OL in LPS-activated BV-2 cells. BV-2 cells were pre-treated for 2 h with 10 µM of OL and OA and then co-exposed to 100 ng/mL of LPS for 24 h. Cell viability was evaluated by MTT assay (**A**), as reported in Materials and Methods. Data are represented as % of CTRL. As reported in Materials and Methods, representative cell morphology images were taken using the microscope Eclipse Ti-E (**B**). Each bar represents means ± SEM of five independent experiments (six units per group). Data were analyzed by one-way ANOVA followed by Tukey’s test. * *p* < 0.05 vs. CTRL; ° *p* < 0.05 vs. LPS.

To ascertain whether the reduction in NO release induced by OL translates into a cytoprotective effect against LPS-induced damage and to assess whether OA, despite not affecting NO levels, might exert a protective effect against LPS-induced damage, we evaluated cellular viability and morphology by MTT assays and microscopy. The data derived from the MTT assay (Figure 3A) revealed a pronounced and significant decrease in cellular viability in cells exposed to LPS. Conversely, pre-treatment with OL completely reversed this effect, increasing cellular viability to levels comparable to those observed in control cells. Notably, the viability of cells pre-treated with OA resembled that of the LPS-exposed group, indicating that OA does not influence this parameter. The cellular morphology, as shown in Figure 3B, reflects the data obtained with the MTT assay. Cells exposed to LPS are visibly fewer in number compared to the control cells, accompanied by the development of an activated phenotype characterized by a rounded cellular morphology. A similar morphological change was observed in cells treated with OA. In contrast, control cells and the OL-treated cells exhibited a comparable “unstimulated” morphology and were visibly more abundant in number. Cumulatively, these findings confirm that only OL effectively mitigates LPS-induced damage.

### 3.4. Gene Expression of Pro- and Anti-Inflammatory Mediators in BV-2 Cells Treated with OL and OA

**Figure 4:**
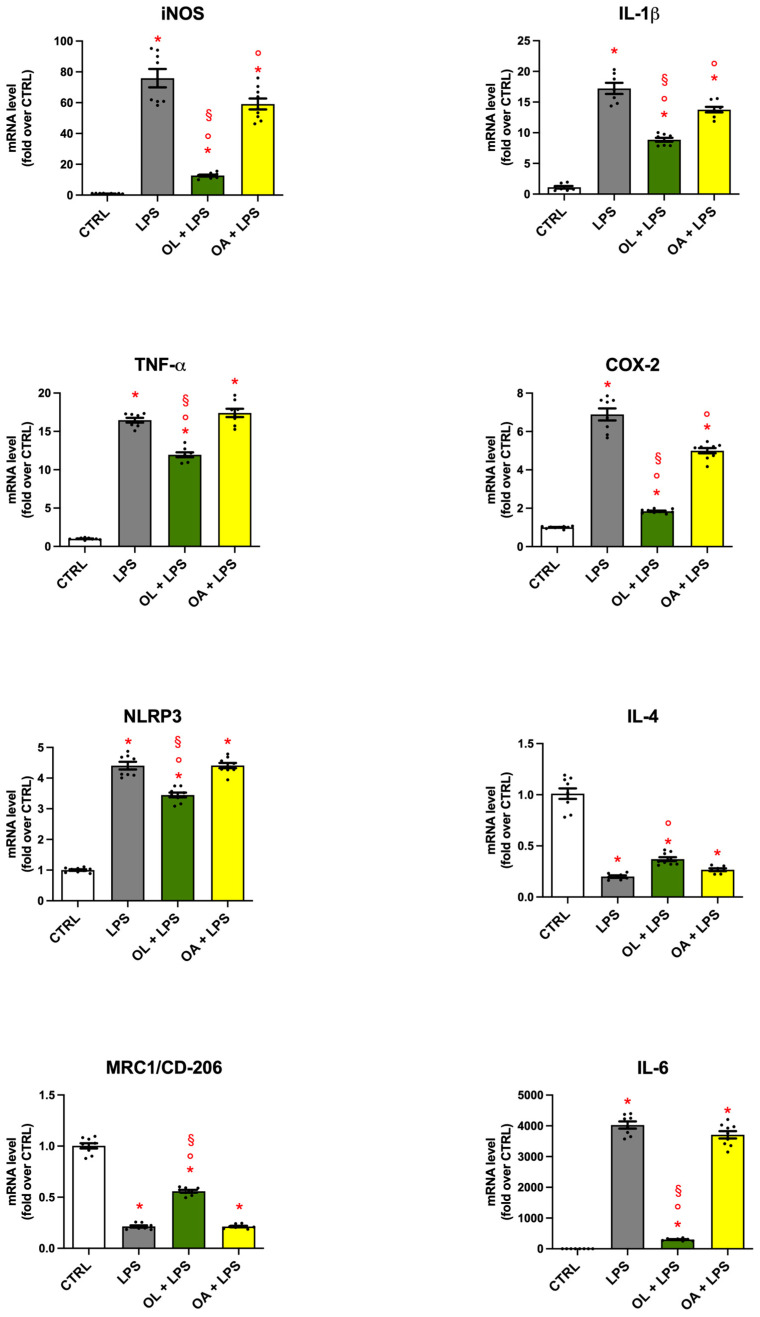
Gene expression of pro- and anti-inflammatory mediators in LPS-treated BV-2 cells. BV-2 cells were pre-treated for 2 h with 10 µM of OL and OA and then co-exposed to 100 ng/mL of LPS for 24 h. mRNA levels were quantified by RT-PCR as reported in Materials and Methods. Data are represented as fold-over control cells. Each bar represents means ± SEM of three independent experiments (at least two units per group). Data were analyzed by one-way ANOVA followed by Tukey’s test. * *p* < 0.05 vs. CTRL; ° *p* < 0.05 vs. LPS; and § *p* < 0.05 vs. OA + LPS.

To investigate the potential impact of OL and OA on inflammatory mediators in LPS-activated BV-2 cells, the mRNA levels of iNOS, COX-2, IL-1β, TNF-α, NLRP3, IL-6, IL-4, and MRC1 were assessed by RT-PCR (Figure 4). As expected, LPS stimulation significantly upregulated the expression of all pro-inflammatory mediators (iNOS, COX-2, IL-1β, TNF-α, NLRP3, and IL-6), and, on the other hand, significantly downregulated the expression of all anti-inflammatory mediators (IL-4 and MRC1), with respect to control cells. OL significantly reduced LPS-induced overexpression of iNOS, COX-2, IL-1β, TNF-α, NLRP3, and IL-6, all associated with the M1 phenotype (pro-inflammatory), alongside a significant increase in the gene expression of IL-4 and MRC1, both markers of the M2 phenotype (anti-inflammatory). Conversely, OA downregulated only the expression of iNOS, COX-2, and IL-1β compared to LPS treatment, but this effect was significantly less pronounced with respect to OL.

### 3.5. Oleocanthal Effect on iNOS, NLRP3, and COX-2 Protein Expression

**Figure 5:**
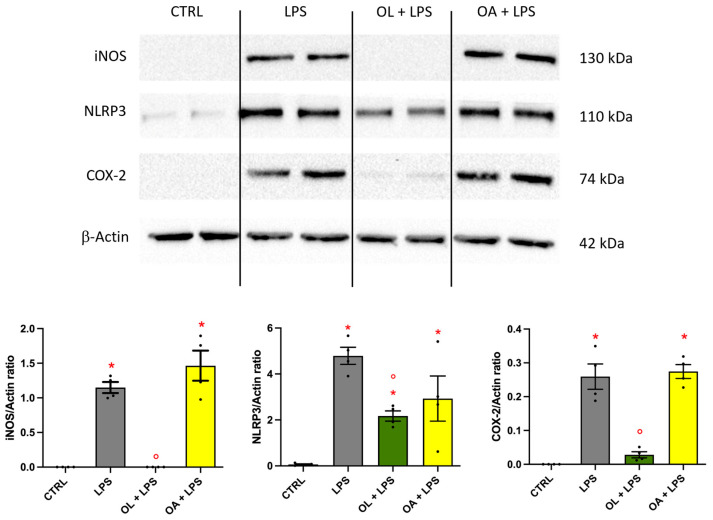
iNOS, NLRP3, and COX-2 protein expression in LPS-treated BV-2 cells. BV-2 cells were pre-treated for 2 h with 10 µM of OL and OA and then co-exposed to 100 ng/mL of LPS for 24 h. Protein expression was analyzed by Western immunoblotting as reported in Materials and Methods. Representative images and densitometric values are shown. The relative bands were normalized to the intensity of the corresponding β-actin band. Each bar represents means ± SEM of four independent experiments (one unit per group). Data were analyzed by one-way ANOVA followed by Tukey’s test. * *p* < 0.05 vs. CTRL; ° *p* < 0.05 vs. LPS.

To further explore the potential anti-inflammatory effect of OL and OA, we analyzed the protein expression of the three main enzymes involved in inflammatory processes, namely iNOS, NLRP3, and COX-2 by Western immunoblotting (Figure 5). Our findings corroborated the observations made through RT-PCR analysis. Specifically, exposure to LPS resulted in a substantial upregulation of all enzymes compared to control cells. Conversely, OL treatment significantly prevented this increase. Notably, and in particular, it was able to maintain the levels of iNOS and COX-2 protein expression comparable with those of control cells. On the other side, OA did not modify the expression of these enzymes.

### 3.6. Oleocanthal Increases Phagocytic Activity in LPS-Stimulated BV-2 Cells

**Figure 6:**
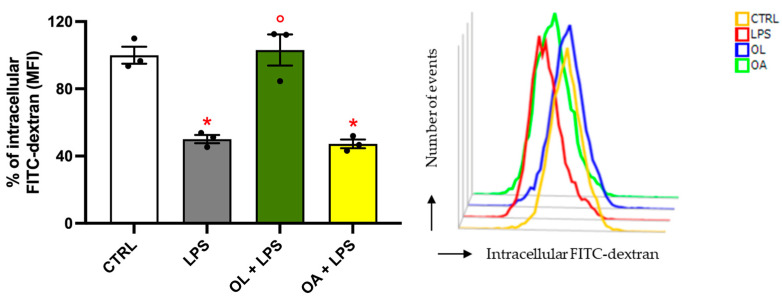
Modulation of the phagocytic capacity by oleocanthal in LPS-activated BV-2 cells. Cells were pre-treated for 2 h with 10 µM of OL and OA and then co-exposed to 100 ng/mL of LPS for 24 h. FITC-dextran fluorescence intensity was determined by flow cytometry, as reported in Materials and Methods. Each bar represents means ± SEM of three independent experiments (one unit per group). Data were analyzed by one-way ANOVA followed by Tukey’s test. * *p* < 0.05 vs. CTRL and ° *p* < 0.05 vs. LPS.

The results from gene and protein expression analyses suggest that, within the neuroinflammation model employed, OL contributes to the polarization of BV-2 cells, promoting a switch from the pro-inflammatory M1 to the anti-inflammatory M2 phenotype. To further substantiate this hypothesis, we tested the phagocytic capacity of BV-2 cells, measuring the median fluorescence intensity (MFI) of FITC-dextran phagocytosed by cells (Figure 6). Phagocytic activity is an important function for immune cells, including microglia, which is related to the resolution of the inflammatory process and, consequently, to the prevalence of the M2 phenotype. As expected, the LPS stimulation significantly decreased the phagocytic capacity of BV-2 cells compared to control cells. Similarly, cells exposed to OA exhibited no alteration in FITC-dextran MFI compared to the LPS group. Once again, OL was demonstrated to completely mitigate the negative effects of LPS exposure, as evidenced by the phagocytic activity of the OL + LPS cells, which was comparable to that of the control cells. These results underscore the significant role of OL in maintaining efficient phagocytosis in BV-2 cells, an established hallmark of M2 polarization.

### 3.7. Antioxidant Activity of Oleocanthal and Oleocanthalic Acid in LPS-Stimulated BV-2 Cells

**Figure 7:**
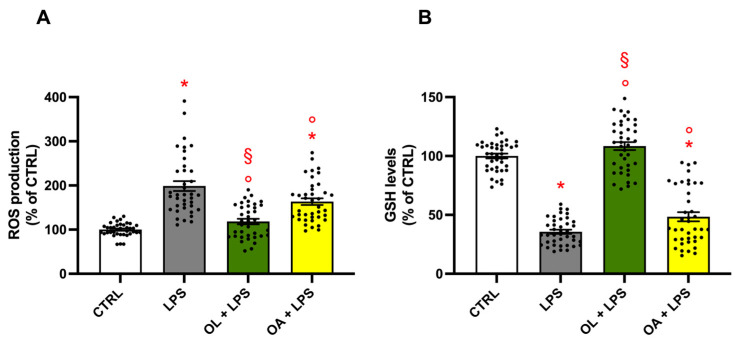
Effect of oleocanthal and oleocanthalic acid on ROS and GSH levels. BV-2 cells were pre-treated for 2 h with 10 µM of OL and OA and then exposed to 100 ng/mL of LPS for 24 h. Intracellular ROS levels were measured using the peroxide-sensitive probe, while DCFH-DA and GSH levels were measured using the fluorescent probe MCB, as reported in Materials and Methods. Each bar represents the mean ± SEM of four independent experiments (10 units per group). Panel (**A**) shows the intracellular ROS levels; Panel (**B**) shows GSH levels. Data were analyzed by one-way ANOVA followed by Tukey’s test. * *p* < 0.05 with respect to CTRL; ° *p* < 0.05 with respect to LPS; and § *p* < 0.05 vs. OA + LPS.

Neuroinflammation is closely associated with oxidative stress, as the M1 phenotype is recognized for its role in the generation of ROS as signaling molecules. These ROSs, in turn, perpetuate and induce the release of inflammatory mediators, thereby establishing a self-feeding vicious circle. To examine whether OL and OA can impact cellular oxidative stress status, we quantified the levels of intracellular ROS (Figure 7A) and reduced GSH (Figure 7B) using spectrofluorimetric assays. As expected, LPS stimulation impaired the redox equilibrium, significantly increasing the production of pro-oxidating agents (Figure 7A) and reducing the tripeptide GSH, the main intracellular antioxidant (Figure 7B). Conversely, treatment with OL effectively restored homeostatic conditions, as indicated by ROS, and reduced GSH levels that were comparable to those observed in control cells. In contrast to the aforementioned findings, OA showed moderate antioxidant activity, significantly reducing ROS production and elevating GSH levels compared to LPS treatment.

### 3.8. NF-κB Transcription Factor, Effect on Its Phosphorylation, and Migration to the Nucleus

**Figure 8:**
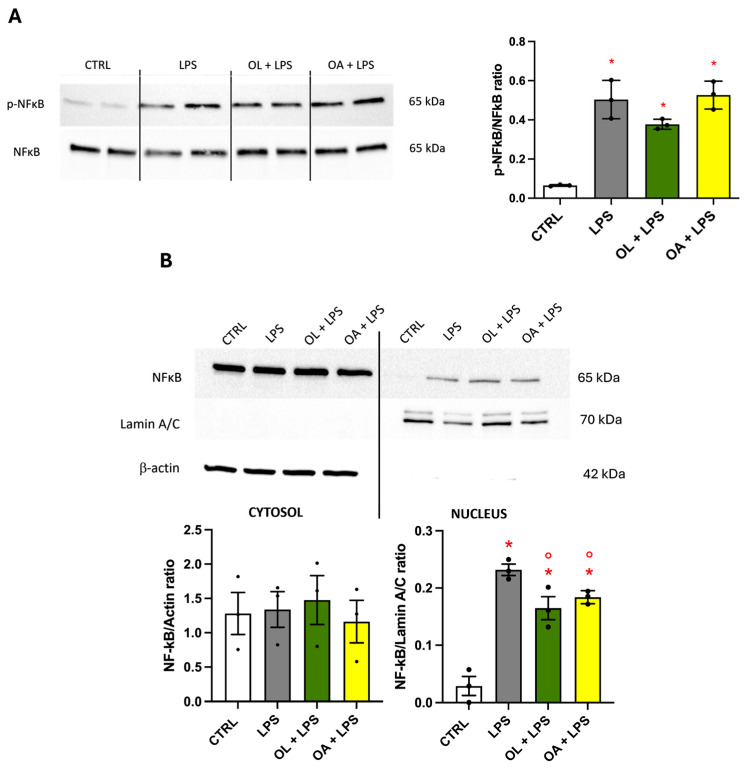
Modulation of NF-κB transcription factor. BV-2 cells were pre-treated for 2 h with 10 µM of OL and OA and then exposed to 100 ng/mL of LPS for 24 h. Samples have been processed for protein expression analysis as reported in Materials and Methods. Each bar represents the mean ± SEM of three independent experiments (one unit per group). Panel (**A**) shows the protein expression of p-NF-κB and NF-κB; Panel (**B**) shows the cytosolic and nuclear expression of NF-κB. Data were analyzed by one-way ANOVA followed by Tukey’s test. * *p* < 0.05 with respect to CTRL and ° *p* < 0.05 with respect to LPS.

The NF-κB transcription factor is a crucial pathway in the M1-activated pro-inflammatory phenotype, controlling the expression of the main inflammatory mediators. For this reason, we wanted to verify if our treatments could influence its activation (phosphorylation) and/or its migration to the nucleus. At the end of the treatments, the cells were lysed and processed for the protein expression analyses, using the total lysate or the samples deriving from cytosol and nucleus separation, by Western immunoblotting. As shown in Figure 8A, cells exposed to LPS significantly increased their levels of phosphorylated NF-κB, while OL slightly reduced this ratio compared to LPS alone, even though this reduction is not significant. At the same time, OA did not change this parameter with respect to LPS. In Figure 8B, we evaluated the migration of NF-κB from the cytosol to the nucleus. As expected, the LPS exposure stimulated a significant elevation in the nuclear levels of NF-κB; unexpectedly, both OL and OA slightly significantly decreased nuclear NF-κB compared to LPS. Simultaneously, no changes have been detected in the cytoplasmic levels of NF-κB.

### 3.9. Analysis of TLR4 and CD14 Surface Expression

**Figure 9:**
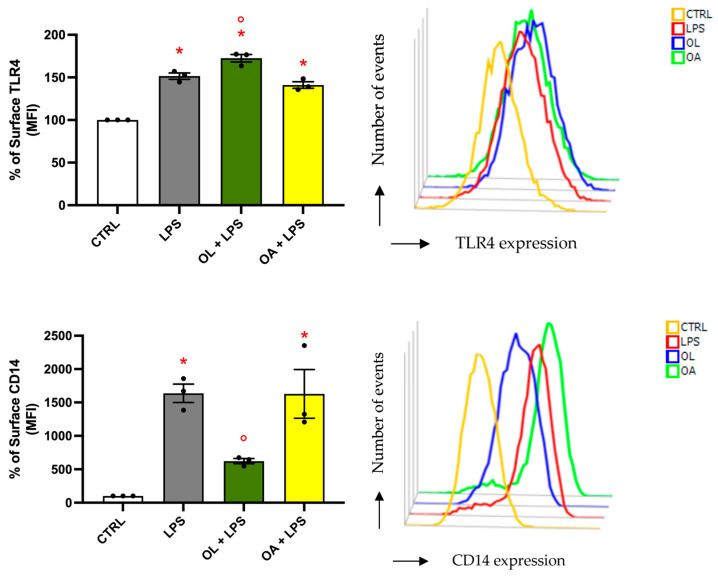
TLR4 and CD14 surface expression of BV-2 cells. BV-2 cells were pre-treated for 2 h with 10 µM of OL and OA and then exposed to 100 ng/mL of LPS for 24 h. Surface expression of TLR4 and CD14 was determined by flow cytometry, as reported in Materials and Methods. Each bar represents the mean ± SEM of three independent experiments (one unit per group). Panel A shows the surface expression of TLR4; Panel B shows the surface expression of CD14. Data were analyzed by one-way ANOVA followed by Tukey’s test. * *p* < 0.05 with respect to CTRL and ° *p* < 0.05 with respect to LPS.

To explore the underlying mechanisms of OL anti-inflammatory activity, we analyzed the expression of the TLR4 receptor on the cell membrane. The TLR4 receptor system is responsible for the initiating of inflammatory and immune responses upon detecting even an infinitesimal amount of circulating LPS, leading to the production of pro-inflammatory mediators [55]. Subsequently, the cells were processed for flow cytometry analyses without the permeabilization step to allow the antibody recognition of the protein present only on the cell surface. As shown in Figure 9 top, LPS activation for 24 h led to a significant increase in TLR4 surface expression with respect to control cells. Similarly, the OA + LPS experimental cells exhibited a comparable increase. Contrary to expectations, OL treatment did not decrease TLR4 presence on the cellular membrane; instead, it led to a significant increase compared to LPS treatment.

This result led us to hypothesize that OL could interfere with the dimerization/endocytosis of TLR4, thus favoring its permanence on the cell surface. To this purpose, we investigate the behavior of the LPS-binding protein CD14, which is also a crucial regulator of TLR4 endocytosis. Cells were treated as previously described, and the surface levels of CD14 were observed by flow cytometry (Figure 9 bottom). In agreement with TLR4 expression on the cell surface, BV-2 cells exposed to LPS exhibited significantly higher CD14 levels compared to control cells. Exposure to OA did not influence CD14 levels relative to LPS; however, treatment with OL dramatically reduced the surface levels of CD14. This reduction of CD14 on the cell membrane may have compromised its dimerization with TLR4, thereby reducing TLR4 endocytosis and potentially explaining TLR4’s higher levels on the membrane in the presence of OL.

### 3.10. Modulation of MAPKs and Protein Kinase Akt Activation

**Figure 10:**
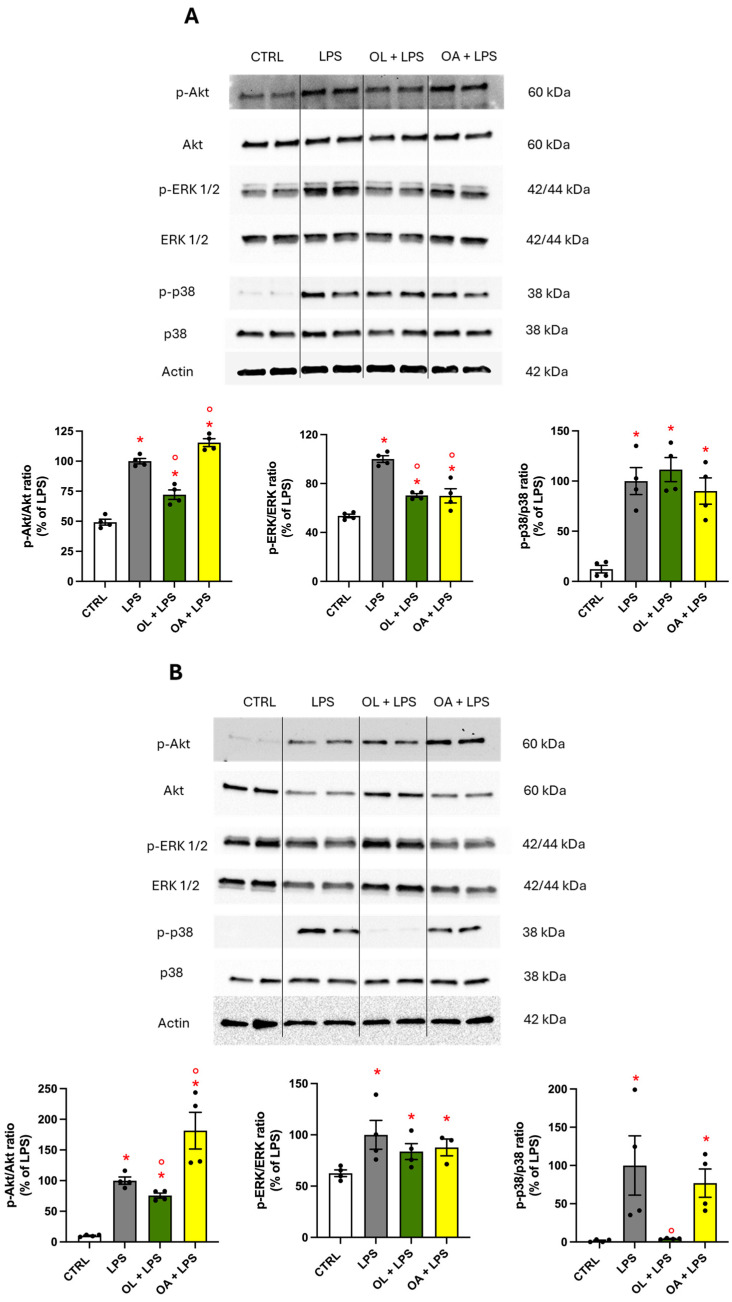
ERK 1/2, p38 MAPKs, and protein kinase Akt modulation by OL and OA on LPS-activated BV-2 cells. BV-2 cells were pre-treated for 2 h with 10 µM of OL and OA and then co-exposed to 100 ng/mL of LPS for 1 h or 24 h. Immunoblotting was performed using the total and the phosphorylated forms of anti-ERK1/2, anti-p38, and anti-Akt. Each bar represents means ± SEM of at least four independent experiments (one unit per group). Panel (**A**) shows the phosphorylation of ERK 1/2, p38 MAPKs, and protein kinase Akt after 1 h of LPS stimulation; Panel (**B**) shows the phosphorylation of ERK 1/2, p38 MAPKs, and protein kinase Akt after 24 h of LPS stimulation. Data were analyzed by one-way ANOVA followed by Tukey’s test. * *p* < 0.05 with respect to CTRL and ° *p* < 0.05 with respect to LPS.

To explore whether the suppressive activity of OL on pro-inflammatory modulators could be mediated by MAPKs and protein kinase-signaling pathways, we evaluated the phosphorylation and, consequently, the activation of ERK 1/2, p38 MAPK, and Akt after 1 h or 24 h of LPS stimulation. BV-2 cells were pre-treated with 10 µM of OL and OA for 2 h prior to the co-treatment with 100 ng/mL of LPS for 1 h (Figure 10A) or 24 h (Figure 10B). The results evidenced a different behavior of kinase activation depending on the timing of LPS exposure. Specifically, after 1 h, LPS exposure significantly increased the phosphorylation of all proteins analyzed compared to control cells. Pre-treatment with OL inhibited the activation of protein kinase Akt and significantly reduced the phosphorylation of MAPK ERK1/2, while pre-treatment with OA decreased the phosphorylation of MAPK ERK1/2 and significantly increased the p-Akt/Akt ratio compared to LPS. In contrast, the activation of MAPK p38 was not altered by either OL or OA. The outcomes were slightly different after 24 h of LPS stimulation. All proteins were phosphorylated and, thus, activated, following LPS exposure compared to control cells. In this scenario, pre-treatment with OL significantly and strongly prevented the phosphorylation of MAPK p38, reducing the levels of p-p38 to those of the control cells. At the same time, the phosphorylation of no other proteins was impacted by OL or OA, with the exception of Akt, whose activation was decreased or increased by pre-treatment with OL or OA, respectively, as compared to LPS alone (Figure 10B).

### 3.11. The Timing of the Treatment Is Crucial for OL’s Anti-Inflammatory Activity

**Figure 11:**
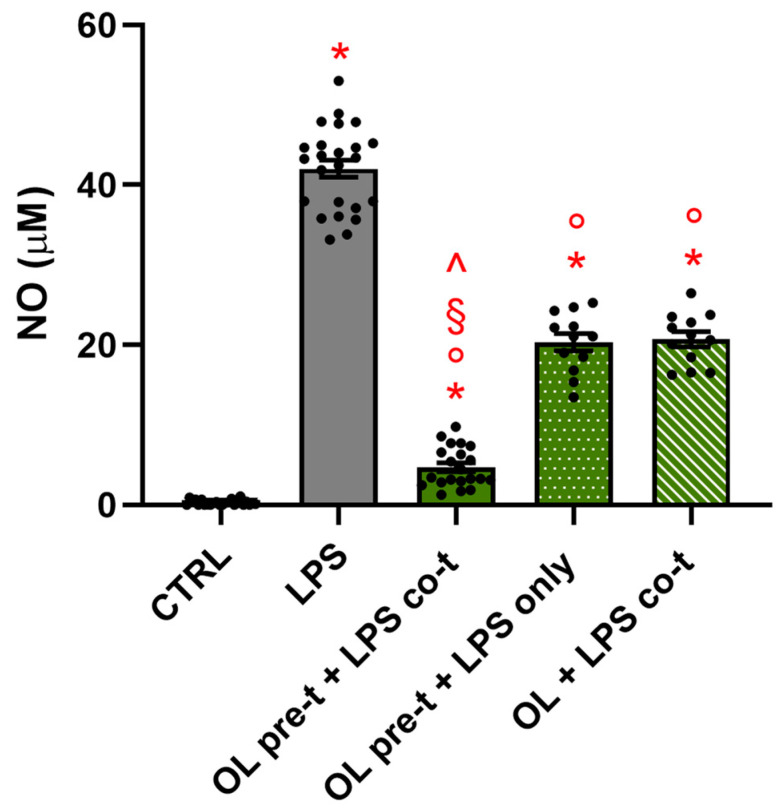
NO release after different timing sets of OL treatment on LPS-stimulated BV-2 cells. BV-2 cells were pre-treated for 2 h with 10 µM of OL, and then 100 ng/mL of LPS were added for 24 h (OL pre-t. + LPS co-t.); BV-2 cells were pre-treated for 2 h with 10 µM of OL, and 100 ng/mL of LPS were added for 24 h after changing the medium (OL pre-t. + LPS only); BV-2 cells were simultaneously exposed to 10 µM of OL and 100 ng/mL of LPS for 26 h (OL + LPS co-t.); NO release was quantified by the Griess reagent. Data are represented as µM of NO released in the culture medium. Each bar represents means ± SEM of three independent experiments (at least four units per group). Data were analyzed by one-way ANOVA followed by Tukey’s test. * *p* < 0.05 vs. CTRL; ° *p* < 0.05 vs. LPS; § *p* < 0.05 vs. OL + LPS co-t; and ^ *p* < 0.05 vs. OL pre-t + LPS only.

The results regarding the levels of TLR4 and CD14 on the cell membrane led us to hypothesize that OL may interfere with the binding of LPS to proteins involved in TLR4-mediated signal transduction. To investigate a potential competition between LPS and OL for receptor binding, we evaluated the effects of either pre-treatment alone or co-treatment alone compared to the previous treatment (pre-treatment + co-treatment). In the first scenario, cells were pre-treated with OL for 2 h, after which the compound was removed, the cells were washed, and LPS was added (thereby excluding potential competition and an effect due to an intracellular activity of OL). In the second case, both compounds were added simultaneously for 26 h to study the extracellular or intracellular activity of OL. The release of NO in the culture medium was used as an indicator of inflammatory activation (Figure 11). It is noteworthy that both in the pre-treatment + LPS only and co-treatment scenarios, there is a significant increase in NO compared to the pre-treatment + combined treatments, indicating a significant reduction in the protective effect. These data suggest that OL likely acts through a pleiotropic mechanism both at intracellular levels (as demonstrated by the effect of the pre-treatment alone) and extracellular levels (evidenced by the fact that the compound’s removal during LPS treatment reduces its protective efficacy).

### 3.12. Proteomic Analysis

**Figure 12:**
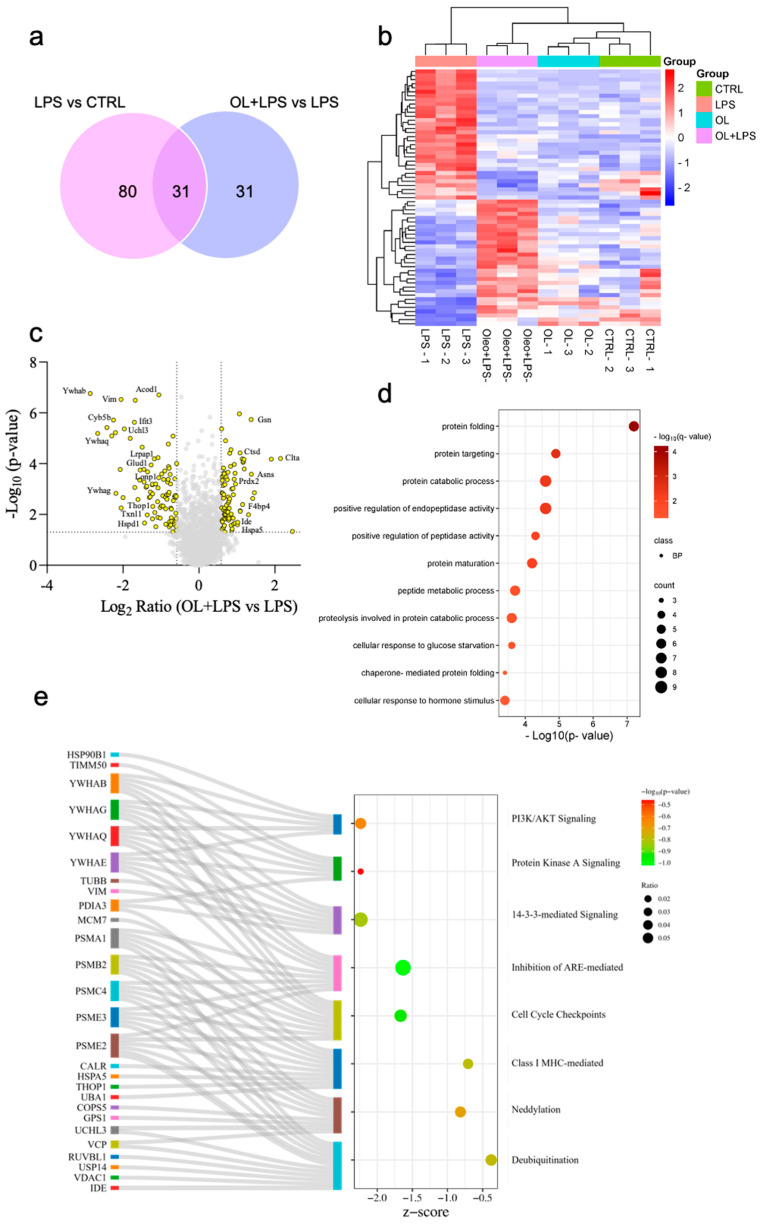
Graphical representation of proteomic results. (**a**) Overlap analysis of differentially expressed proteins in mouse BV-2 cells treated with LPS in the presence or absence of OL. (**b**) Heatmap of proteins identified in OL + LPS vs. LPS comparison (n = 3). Data analyzed by RStudio and the row clustering distance and clustering method were “euclidean” and “complete”, respectively. (**c**) Volcano plot of quantified proteins obtained for OL + LPS vs. LPS comparison. Colored points represent differentially expressed proteins with *p*-value < 0.05 and fold ≥1.5. Dotted lines indicate the threshold of significance and fold values. The gene names of identified proteins are shown in the scatter plot. (**d**) Enrichment bubble plot of GO biological processes. Size of circle for each biological process represents counts of enriched proteins. (**e**) Sankey diagram and bubble plot of canonical pathways in OL + LPS vs. LPS comparison. The enriched canonical pathways are listed based on the IPA analysis. The size of circle for each pathway represents the ratio, whereas the color is the significance. The ratio is calculated from the number of molecules in a particular pathway divided by total number of molecules that make up that pathway and that are in the reference set. In x axis of bubble plot negative, z score values of canonical pathways are achieved in OL + LPS treatment.

To better clarify this complex mechanism of action of OL against LPS-induced damage in microglial cells, we conducted a quantitative proteomic analysis of protein extracts from BV-2 cells under various treatment conditions (control, LPS, and pre-treatment with OL for 2 h, followed or not by co-treatment with LPS for 24 h). The results obtained from the comparison of 2DE images showed that exposure to LPS led to a significant change in the expression of 111 protein spots compared to control, demonstrating the efficacy of LPS activation (Figure 12a). Of these, 31 spots were differentially expressed in the OL + LPS vs. LPS comparison. Interestingly, in OL-treated cells, these proteins showed expression levels comparable to control cells. Overall, 62 protein spots were differentially expressed in the OL + LPS vs. LPS comparison (Figure 12a). These spots were identified by LC-MS/MS. The names of the identified proteins, the molecular weight (MW), isoelectric point (pI), score, coverage values of MS/MS, ratio, and *p* values are listed in Table S1 (Supplementary Materials). Heatmap of differentially expressed proteins in OL + LPS vs. LPS comparison shows how OL was able to counteract the protein modulation induced by LPS activation. Interestingly, OL treatment in the absence of LPS did not result in substantial modifications of the protein expression pattern observed in control cells (Figure 12b). A Volcano plot was constructed to represent fold change and *p*-value in protein expression for this comparison. Gene names of proteins that exhibited the greatest increase and decrease in expression were indicated in the plot (Figure 12c). Notably, OL reversed the inhibitory effect of LPS on proteins such as gelsolin, while it also reduced the significant increase of expression, induced by LPS, of proteins such as 14-3-3β, τ, ε, and γ.

To elucidate the effects of OL on BV2 microglia cells stimulated with LPS, we conducted a protein enrichment analysis on our proteins dataset. Among biological processes identified, there was a predominant involvement of protein functions. Of note, we observed protein stimulus, protein folding, protein catabolic process, regulation of protein stability, and cellular response to cytokines stimulus (Figure 12d). Using IPA software (IPA, QIAGEN, Redwood City, CA, USA, www.qiagen.com/ingenuity, Content version: 21249400, accessed 24 August 2023), we analyzed differentially expressed proteins in the OL + LPS versus LPS comparison, which were associated with canonical pathway features. Overall, OL counteracted the activation of PI3k/AKT signaling, protein kinase A signaling, 14-3-3-mediated signaling, cell cycle checkpoint, and neddylation, caused by LPS stimulation. Figure 12e depicts a Sankey diagram in which canonical pathways are correlated with proteins that were found to be differentially expressed. Meanwhile, the bubble graph displays z-score values and *p*-values obtained from the analysis of the OL + LPS condition. Overall, these data demonstrate a profound impact of OL on the signaling pathways of microglia in response to LPS-induced inflammation.

### 3.13. Analysis of Molecular Docking Results

Based on the results on TLR4 and CD14 surface expression, suggesting that OL may interfere with the binding of LPS to TLR4 and its subsequent endocytosis, we conducted a docking analysis to evaluate a potential interaction of OL with CD14 or MD2. These two proteins are TLR4 coreceptors that bind LPS and facilitate TLR4 dimerization and downstream signaling [56]. In addition to delivering LPS to surface TLR4-MD2, CD14 can mediate LPS-induced endocytosis of TLR4 [57].

**Figure 13:**
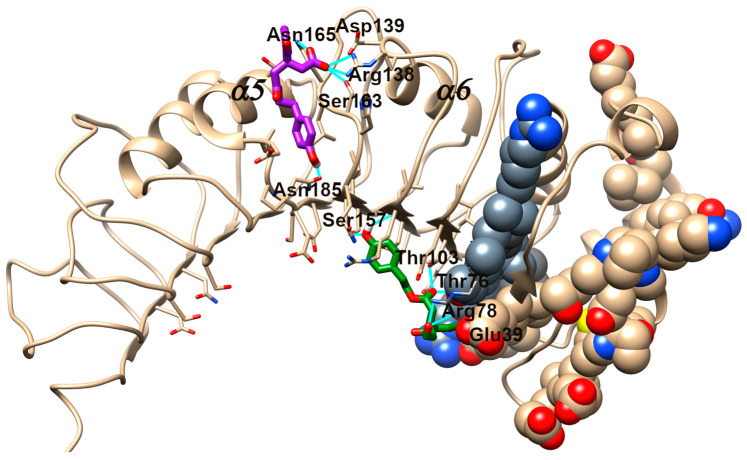
Molecular docking results of OL and OA (green and purple respectively) in *m*CD14 (PDB ID 1WWL). Residues detected through mutagenesis studies to be responsible for LPS binding and LPS signaling are shown in tan and gray spheres, respectively. Residues involved in the binding of OL and OA are highlighted.

The crystal structure of mCD14 (PDB ID: 1WWL [48], see Figure 13) revealed a very large pocket with a flexible rim, and both the pocket area responsible for LPS binding and the regions responsible for LPS signaling have been intensively studied by mutagenesis experiments. In Figure 13, a schematic representation of residues involved in LPS binding (tan spheres) and in LPS signaling (grey spheres) is reported. Nevertheless, despite the high structure similarity between OL and OA, the blind docking results of these compounds in CD14 are completely different. The presence of carboxylate directs OA in the loop region between α5 and α6, where interactions with Arg138, Ser163, Asn165, and Asn185 fix the molecule on the surface. Diversely, OL preferably occupies the β-sheet region through interactions with Thr103, Ser157, Thr76, and Arg78, which are in the region responsible for LPS signaling. The α, β-unsaturated carbonylic moiety reaches Glu39 in the adjacent LPS-binding region. This docking mode could interfere with the usual LPS cascade.

**Figure 14:**
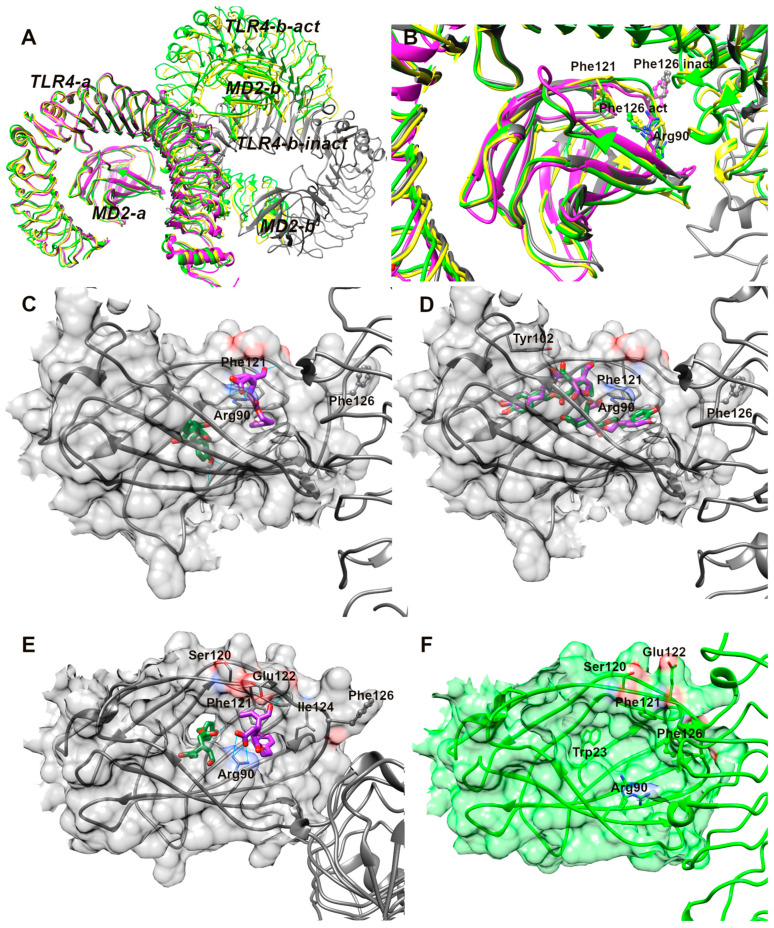
TLR4-MD2 complex structures in different conformations, relative to activated state (green and yellow) and inactive state (black and magenta). (**A**) Superposition of mTLR4-MD2-lipidA (green, PDB ID: 5IJD), mTLR4-MD2-neoseptin-3 (yellow, PDB ID: 5IJC), ligand-free mTLR4-MD2 (black, PDB ID: 5IJB), and TLR4-TV3 hybrid-MD2-Eritoran (magenta, PDB ID: 2Z65) complexes. (**B**) Detailed view of the MD2 core inside the complex highlighted the conformation of Phe126 in the activated state (inner pose, green, and yellow) and inactive state (outer pose, black, and magenta). (**C**) Front view of the best docking poses of oleocanthalic acid (purple) and oleocanthal (green) in the inactive form of mMD2 (PDB ID: 5IJB). (**D**) Alternative docking pose of oleocanthalic acid (purple) and oleocanthal (cyan) in the inactive form of mMD2 (black, PDB ID: 5IJB). Hydrogen bonds are cyan-colored. (**E**) Top view of the best docking poses of oleocanthalic acid (purple) and oleocanthal (green) in the inactive form of mMD2 (PDB ID: 5IJB). (**F**) Top view of the activated form (via lipidA interaction) of mMD2 (PDB ID: 5IJC). Residues with high conformational variability during the activation are highlighted.

A second hypothesis is that OL could interfere with the activation/dimerization of TLR4, thus favoring its permanence on the cell surface through the interaction with MD-2. A recent publication on the mouse TLR4—MD-2 complex reveals that the apo form of mTLR4/MD-2 is a monomeric 1:1 complex that adopts an inactive dimer conformation in the crystal (black colored in Figure 14A), in which the contact zone between the two monomeric complexes is different and smaller in size than in the activated one (green and yellow colored in Figure 14A) [47].

The same inactive local conformation is found at the interface between MD-2 and TLR4 in the human TLR4 TV3 hybrid-MD-2 (magenta colored in Figure 14) in complex with Eritoran, a TLR4 antagonist [58]. Otherwise, both lipid A and the agonist Neoseptin-3 induce the formation of an activated dimer (green and yellow colored complexes in Figure 14A) consisting of two mTLR4/MD-2 heterodimers arranged symmetrically in an “m” shape (as observed in the structures of TLR4/MD-2 bound to LPS [59] reported in the literature), which share a similar local conformation in MD-2 around the bound ligand and at the interface between the two mTLR4/MD-2 heterodimers. In particular, the MD-2 loop region containing Phe126 (ball and stick represented in Figure 14) undergoes a conformational change in both complexed structures of lipid A and Neoseptin-3 (yellow and green in Figure 14B). In the activated form, the aromatic ring of Phe126 faces inward towards MD-2, while in the inactive structure, Phe126 faces towards TLR4 (Figure 14B). It is not known how this conformational change is correlated to the activation of the TLR4 cascade, but it is structurally validated.

Our computational study on OL and OA detected three principal poses; the first one is reported in Figure 14C and the alternatives are reported in Figure 14D. The latter two are very similar for both OL and OA: both compounds are inserted into the hydrophobic cavity, in the half cavity of Tyr102, or in the opposite region of Arg90. Both residues are highlighted as critical by mutagenesis studies [60]. Here, OL and OA are stabilized only by lipophilic interactions, without particular structural details that can rationalize the difference in their behavior in biological tests.

The poses reported in Figure 14C reveal very different binding modes in the MD-2 site. OL is deeply inserted in the pocket, pointing the phenolic -OH towards the bottom of the cavity, while OA is attached to the rim of the cavity through an ionic bond between its carboxylate and Arg90. So, OL is able to insert in the inner part of the MD-2 cavity, while OA lies at the entrance. Furthermore, comparing the inactive and activated conformations of MD-2 (see Figure 14E,F), a hole at the bottom of the cavity in the former, where OL points its phenolic ring (Figure 14E), is detectable, while the activation process seems to close the hole by Trp23 in the mTLR4-MD2-lipidA complex (Figure 14F). This region is highly variable in the available 3D structures, and it has recently been identified as a potential binding site for heme [61]. The insertion of OL in the bottom “tunnel” of MD-2 could interfere with the insertion of LPS and/or prevent some conformational changes at the bottom of the cavity crucial for the activation/dimerization of TLR4. The alternative pose of OL reported in Figure 14D, filling the core of the MD-2 cavity, could compete with the direct binding of LPS. Instead, the OA, in the predicted location right at the entrance of the protein, could be easily displaced in the presence of LPS.

## 4. Discussion

Neurodegenerative diseases, including Alzheimer’s disease, Parkinson’s disease, and multiple sclerosis, represent a significant challenge due to their multifactorial pathophysiology and the absence of drugs capable of halting the progression of these diseases. One of the key pathological hallmarks common to these diseases is neuroinflammation, which has been extensively documented as playing a crucial role in their progression [62,63].

Neuroinflammation is characterized by the activation of glial cells, particularly microglia, which are the resident immune cells of the central nervous system [64]. While acute inflammation can be protective for the brain, chronic neuroinflammation is detrimental and contributes to neuronal damage and the progression of neurodegenerative diseases.

Modulation of microglial activity represents a promising strategy to counteract neurodegeneration. Recent studies have highlighted various natural compounds that can reduce neuroinflammation both in vivo and in vitro [39,65,66,67]. Among these compounds, OL, a phenolic compound found in EVOO, has emerged as a potential anti-inflammatory agent. In vitro, OL has been shown to inhibit the activity of the inflammatory enzymes COX-2 and COX-1, exhibiting characteristics remarkably comparable to those of ibuprofen [68]. However, the stability and activity of OL during the storage of EVOO have raised questions about its long-term effectiveness. During storage, oleocanthal undergoes non-enzymatic oxidation, forming OA [26]. This transformation poses the question of whether OA retains the anti-inflammatory properties of OL or if it plays any significant role in fighting neuroinflammation.

In our study, we investigated the anti-inflammatory properties of OL and its oxidation product, OA, using the murine microglial cell line BV-2 activated with LPS as an in vitro model of neuroinflammation. This experimental model is an effective and widely used tool for studying neuroinflammation in the context of neurodegenerative diseases like AD and PD [69]. This model is valuable because LPS is known to mimic bacterial infection by activating microglia through the toll-like receptor 4 (TLR4), which triggers a cascade of inflammatory responses. In particular, it has been observed that LPS levels in the blood of AD and PD patients are increased [70,71,72,73,74], and the injection of elevated levels of LPS into the blood of healthy humans leads to cognitive dysfunction [75,76]. 

Our results demonstrate that OL significantly reduced neuroinflammation by modulating the expression of key cytokines and inflammatory enzymes, while OA showed only a marginal effect. Specifically, OL treatment completely counteracted LPS-induced cell death, significantly reduced NO release, decreased the levels of pro-inflammatory mediators such as TNF-α, IL-1β, NLRP3, IL-6, COX-2, and iNOS, upregulated the expression of the anti-inflammatory mediators like IL-4 and MRC1, and increased BV-2 cell phagocytic activity compared to LPS-treated cells, suggesting a shift from the M1 to M2 phenotype.

The increase in phagocytic activity is crucial in the context of neurodegenerative diseases such as Alzheimer’s disease. Microglia play a key role in maintaining homeostasis by clearing amyloid-β aggregates through phagocytosis [77,78], and their inability to remove amyloid plaques in late disease has been implicated as a cause of accelerated neurodegeneration [79]. Based on these considerations, OL could play a role in counteracting the accumulation of amyloid plaques.

Furthermore, OL was able to counteract LPS-induced oxidative stress by drastically reducing ROS production and significantly increasing GSH levels. On the other hand, OA was unable to modulate any of the aforementioned parameters, except for a slight reduction in the expression levels of iNOS, COX-2, and IL-1β, as well as a modest modulation of the cellular redox state, which did not translate into actual protection as measured by the MTT assay. The antioxidant activity of OA aligns with previous findings, where using in vitro tests, we demonstrated that it possesses an O_2_^•−^ scavenging capacity comparable to that of ascorbic acid [30]. It is likely that the lower capacity of OA to counteract LPS-induced oxidative stress compared to OL is due to the fact that it acts through a direct antioxidant mechanism, unlike OL, which also acts indirectly by countering neuroinflammation.

In light of the promising results of OL against neuroinflammation, and considering that, to the best of our knowledge, no study has investigated the anti-inflammatory activity of this compound in microglial cells, we decided to shed light on the mechanism of action of OL, also using OA to characterize their different activity.

Contrary to our expectations, while we observed a reduction in NF-κB phosphorylation and nuclear translocation with OL treatment, the magnitude of this reduction was not as pronounced as we had assumed. Moreover, when comparing the degree of NF-κB nuclear translocation in response to LPS, both OL and OA exhibited comparable reductions. This similarity was unexpected, particularly because it does not adequately explain the strong different effects that these compounds have on the modulation of inflammatory mediators, suggesting that the mechanisms underlying the anti-inflammatory effects of OL are more complex than initially thought. In the context of neuroinflammation, the modulation of specific kinases such as Akt, ERK, and p38 plays a crucial role [80,81,82]. The comparative analysis of OL and OA on the Akt, ERK, and p38 modulation reveals that OL is significantly more effective in counteracting the activation of these proteins. The complete inhibition of p38 phosphorylation by OL suggests that this kinase could be a critical molecular target for OL in exerting its anti-inflammatory activity. Of note, OL is not able to reduce p38 activation at 1 h LPS exposure, while it significantly decreases its activation after 24 h of LPS. This suggests that OL does not counteract the mechanisms leading to the phosphorylation of this kinase but rather seems to upregulate the mechanisms responsible for turning off this signal. One hypothesis is that OL could trigger the dephosphorylation of phospho-p38. A very recent study shows that the upregulation of MKP-1, a phosphatase that dephosphorylates MAPKs such as JNK, ERK1/2, and p38, can counteract neuroinflammation, reducing MAPK activation and promoting the M2 phenotype [83]. In our case, OL most likely does not act on MKP-1, as we would have observed a deactivation effect on phospho-ERK as well. OL might be acting by upregulating a specific phosphatase that selectively targets phospho-p38. To the best of our knowledge, no phosphatase has yet been identified that acts selectively on phospho-p38. Of course, other mechanisms could be involved in turning off the p38 signal. This aspect is certainly of primary importance and deserves further investigation.

Another possible mechanism by which OL could counteract neuroinflammation is through the modulation of LPS binding to TLR4. TLR4 is one of the most thoroughly researched proteins within the TLR family and is the canonical receptor for LPS [84]. LPS binds to TLR4 with the assistance of an LPS-binding protein and CD14, which may trigger the MyD88-dependent- and the TRIF-dependent-signaling pathways, leading to the release of proinflammatory mediators and contributing to neuroinflammation [85]. CD14 aids in the clustering and internalization of the receptor complex into endosomes, a critical process for the subsequent activation of downstream signaling pathways [85]. We observed an unexpected increase of TLR4 on the cell membranes in OL-treated cells compared to LPS, while CD14 levels were strongly reduced by OL. On the other hand, OA was not able to counteract the effects of LPS on either TLR4 or CD14, as it displayed values comparable to those triggered by LPS. The level of CD14 on the cell surface has been shown to significantly influence TLR4 signaling in macrophages and dendritic cells, as the inflammatory response was reduced when CD14 was deficient and increased when CD14 was abundant [86]. Due to these results, our hypothesis is that, by reducing CD14 levels, OL impairs the internalization of TLR4, leading to an accumulation of TLR4 on the cell membrane without successfully transducing the signal into the cell, thereby reducing the production of inflammatory mediators. One possible explanation for the strong reduction of CD14 on the cell membrane is that OL could directly interact with CD14, causing its internalization. This hypothesis was already explored for a synthetic Lipid A mimetic [87] and for MMPP ((E)-2-methoxy-4-[3-(4-methoxyphenyl) prop-1-en-1-yl] phenol) [88]. On the contrary, OA is unable to promote the endocytosis of CD14 and, as a consequence, probably unable to bind it. Indeed, the results of the docking analysis indicated that, despite the high structure similarity between OL and OA, the two compounds could bind to different CD14 regions, and only OL interacts with aminoacids which are in the region responsible for LPS signaling. These results are speculative at the moment, but they are compatible with our biological results and provide a first rationale. Despite the uncertain mechanism of action, the molecular docking results demonstrate that the presence of the carboxyl moiety of OA can drive a different binding mode, with respect to OL, with CD14. CD14 has charged surfaces that can capture OA and prevent it from entering the internal or critical regions of the protein. In contrast, OL can detect internal cavities.

Another hypothesis is that OL could interfere with the activation/dimerization of TLR4, thus favoring its permanence on the cell surface through the interaction with MD-2, a protein stably associated with the extracellular fragment of TLR4 and essential for LPS binding [85]. This hypothesis was already suggested for natural products like curcumin [89], sulforaphane [90], and chalcone derivatives [91], leading to the identification of different types of interaction in the core. The molecular docking analysis identified three principal binding poses within the MD-2 site. Two poses show a similarity in the binding mode for both OL and OA, where they are located within the hydrophobic cavity near Tyr102 or the opposite region of Arg90. In these poses, OL and OA are stabilized mainly through lipophilic interactions without significant structural details to explain their different biological behaviors. In the third pose, OL is deeply inserted into MD-2 hydrophobic cavity, while OA is situated at the cavity’s entrance. This distinct positioning suggests OL’s potential to interfere with LPS insertion or prevent conformational changes necessary for TLR4 activation, as OL occupies the inner part of the cavity, while OA, being at the entrance, is easily displaceable by LPS. Comparing the inactive and activated conformations of MD-2, a notable feature is the hole at the bottom of the cavity in the inactive state, where OL points its phenolic ring. The activation process closes this hole, suggesting a possible interference by OL with LPS binding or crucial conformational changes for TLR4 activation. These findings reinforce the potential of OL to disrupt TLR4 signaling.

The inhibition of TLR4 signaling by OL alone cannot fully account for the substantial inhibitory effect of this compound on neuroinflammation. Indeed, OL only partially reduces the nuclear translocation of NF-κB, suggesting that the compound might also act on other molecular targets modulated by LPS. To further explore this hypothesis, we conducted a proteomic analysis, which yielded intriguing data regarding both the molecular targets of LPS and the mechanism of action of OL in the context of neuroinflammation. In this study, we have chosen not to extensively discuss the proteins affected by LPS. Our main focus is to evaluate the anti-inflammatory potential of OL and OA, not to characterize the LPS mechanism of action in microglial cells. Nonetheless, the fascinating outcomes reveal that new molecular targets of LPS warrant further exploration. Therefore, these findings will be thoroughly examined in a subsequent study.

By examining the proteomic profiles of untreated cells, OL-treated cells, and cells exposed to LPS in the presence or absence of OL, we identified several key proteins and pathways that are differentially regulated. In particular, proteomic data indicate that LPS modulates 111 proteins compared to control cells, and 31 of these proteins are also modulated by OL. Remarkably, OL restores the levels of these proteins to values comparable to those of control cells. A particularly interesting result from our study is that OL alone does not modulate protein expression. This observation suggests that the effects of OL on neuroinflammation are contingent upon the presence of an inflammatory stimulus, such as LPS.

Of particular interest is the strong and significant downregulation induced by OL compared to LPS of four proteins belonging to the 14-3-3 protein family: the 14-3-3β, τ, ε, and γ isoforms, which are encoded by the YWHAB, YWHAQ, YWHAE, and YWHAG genes, respectively, in humans. The 14-3-3 protein family is highly present in the brain and has been associated with neurodegenerative diseases. Higginbotham et al. observed an increased expression of 14-3-3β, τ, ε, and γ in the cerebrospinal fluid (CSF) of AD patients compared to control subjects [92]. Similarly, another study by Zhou et al. identified elevated levels of 14-3-3β, ε, and γ [93]. Increased levels of the γ-isoform have been reported in AD brain tissue and CSF [94,95,96,97]. In a recent study, Peng et al. observed an upregulation of 14-3-3γ in the serum of PD patients with MCI and Dementia compared to PD without cognitive impairment, suggesting this protein is a biomarker of cognitive impairment in PD patients [98]. From immunohistochemical studies, different 14-3-3 isoforms that are also present in Lewy bodies emerged [94]. The ability of OL to reduce the level of different proteins belonging to the 14-3-3 protein family represents an important aspect in the context of neurodegenerative diseases such as AD and PD, where the levels of these proteins are upregulated. However, future studies are needed to clarify the exact role of these proteins in the onset and progression of neurodegeneration.

Proteomic analysis also evidenced a significant reduction of clathrin light chain A in LPS-treated cells compared to controls, suggesting its downregulation as a consequence of neuroinflammation. Interestingly, OL was able to markedly increase clathrin light chain A in OL-treated cells compared to LPS. Clathrin is crucial for cell signaling by regulating receptor trafficking and is essential for neuron-specific functions like synaptic vesicle recycling and neurotransmitter-receptor trafficking [99]. Inactivation of the clathrin light chain at neuromuscular junctions in Drosophila blocked synaptic vesicle reformation, indicating the necessity of the light chain despite the presence of clathrin-independent internalization mechanisms [100]. Similar results were observed in clathrin light chain knockout mice, which showed electrophysiological defects due to impaired synaptic vesicle recycling [101]. A recent study involving a mouse microglial cell line exposed to fibrillar Aβ1-42 observed that clathrin was found to colocalize with internalized Aβ42. Additionally, inhibiting clathrin-mediated endocytosis (CME) led to an 80% reduction in the uptake of Aβ1-42 [102]. A decrease in CME could potentially impair the clearance of Aβ1-42 by microglia, thereby contributing to neurotoxic effects. Moreover, clathrin plays a critical role in modulating TLR4 and CD14, as it is involved in the endocytosis and trafficking of these receptors [85], which can influence the downstream inflammatory signaling pathways, highlighting its importance as a potential molecular target to modulate neuroinflammation.

Another intriguing protein whose LPS-induced downregulation is reverted by OL is gelsolin (GLS). GLS is a major actin filament regulatory protein that controls cytoskeletal assembly and disassembly, cell motility, cell growth, and apoptosis [103]. In agreement with our results, Cheng et al. showed that LPS reduced GLS levels both in macrophage cell line RAW264.7 and in mice [104]. They also observed that recombinant GLS inhibited the cytokines induced by LPS and rescued mice from LPS-induced death, and si-GLS increased death in the LPS-pretreated mice, suggesting an anti-inflammatory role of this protein. In addition, anti-amyloidogenic properties have been associated with GLS. GLS binds to Aβ under normal physiological conditions, sequestering it, inhibiting its fibrillation, and reducing amyloid load through defibrillation of preformed peptide fibrils [103]. Due to these characteristics, GLS has been suggested as a potential therapeutic target for AD [105].

Another intriguing result from proteomic analysis is the ability of OL to counteract the strong upregulation of aconitate decarboxylase 1 (ACOD1) induced by LPS. ACOD1 catalyzes the synthesis of itaconate, a metabolite produced from the tricarboxylic acid (TCA) cycle intermediate cis-aconitate. Under resting conditions, itaconate is scarcely detectable [106]. However, the levels of both ACOD1 and itaconate significantly increase in immune cells, primarily macrophages, as well as in non-immune cells, in response to pro-inflammatory and oxidative conditions [107,108]. In agreement with our findings, recent studies have reported that upregulated ACOD1 is involved in the initiation and activation of microglia [108]. A recent review by Wu et al. has highlighted that the emerging function of ACOD1 in inflammation and infection is a double-edged sword [109]. The activation of ACOD1 at the transcriptional level leads to the production of itaconate, which can have either anti-inflammatory or pro-inflammatory effects depending on the context, thus playing a role in either preventing or promoting inflammation. Although research on the ACOD1 protein is still in its early stages, further understanding of the ACOD1/itaconate pathway in neurodegeneration might identify a new molecular target for inhibiting neuroinflammation, a common characteristic of neurodegenerative diseases such as AD and PD.

## 5. Conclusions

In conclusion, the comparative analysis between OA and OL demonstrated that when OL is oxidized to OA, it completely loses its anti-neuroinflammatory action, highlighting the importance of maintaining the integrity of nutraceutical compounds in foods. Additionally, the data demonstrate that OL possesses an anti-neuroinflammatory activity that exceeds the mere inhibition of COX-2 activity. Specifically, it significantly reduces inflammatory agents such as IL-1β, TNF-α, IL-6, COX-2, NLRP3, and iNOS, while inducing anti-inflammatory mediators, such as IL-4 and MRC1/CD206, and enhancing the phagocytic activity of BV-2 cells. These characteristics suggest a shift towards the M2 phenotype. Docking analyses suggested a plausible interaction of OL with CD14 and MD-2, which are co-receptors for TLR4. This interaction potentially interferes with TLR4-signaling pathways in an antagonistic fashion. However, since OL only partially reduced the LPS-induced activation of NF-κB, its role as a TLR4 antagonist alone does not fully explain its anti-inflammatory effect. Proteomic analysis revealed that OL counteracts the LPS modulation of 31 proteins, including significant targets such as gelsolin, clathrin, ACOD1, and four different isoforms of 14-3-3 protein, whose role in neurodegeneration has been already demonstrated. In conclusion, since neuroinflammation is a common feature of neurodegenerative diseases, OL could play a crucial role in counteracting conditions such as AD and PD. Moreover, its ability to exert its effects through multiple pathways and mechanisms is of primary importance in the context of neurodegenerative diseases that have a multifactorial etiology.

## Figures and Tables

**Table 1:** Primers used for RT-PCR.

Gene	Forward	Reverse	RefSeq Accession No
*iNOS*	CCTCCTCCACCCTACCAAGT	CACCCAAAGTGCTTCAGTCA	NM_010927
*COX-2*	TGGGGTGATGAGCAACTATT	AAGGAGCTCTGGGTCAAACT	NM_011198
*NLRP3*	GATGCTGGAATTAGACAACTG	GTACATTTCACCCAACTGTAG	NM_145827
*TNF-α*	CCCCAAAGGGATGAGAAGTTC	CCTCCACTTGGTGGTTTGCT	NM_013693
*IL-1β*	GTTCCCATTAGACAACTGCACTACAG	GTCGTTGCTTGGTTCTCCTTGTA	NM_008361
*IL-6*	GTCTATACCACTTCACAAGTC	TGCATCATCGTTGTTCATAC	NM_031168
*IL-4*	CTGGATTCATCGATAAGCTG	TTTGCATGATGCTCTTTAGG	NM_021283
*MRC1*	GTTATGAAAGGCAAGGATGG	ATCAGTGAAGGTGGATAGAG	NM_008625
*GAPDH*	ACCACAGTCCATGCCATCAC	TCCACCACCCTGTTGCTGTA	NM_001289726

*iNOS*: inducible nitric oxide synthase; *COX-2*: cyclooxygenase-2; *NLRP3*: Nod-like receptor protein 3; *TNF-α*: tumor necrosis factor-alpha; *IL-1β*: interleukin-1 beta; *IL-6*: interleukin-6; *IL-4*: interleukin-4; *MRC1*: mannose receptor C-type 1; *GAPDH*: glyceraldehyde-3-phosphate dehydrogenase.

## Data Availability

The data are available on request from the authors.

## Supplementary Materials

The following supporting information can be downloaded at: https://www.mdpi.com/article/10.3390/antiox13091074/s1, Table S1: List of proteins found differentially expressed in the comparison of microglia cells treated with OL + LPS vs. cells treated with LPS.
