# Supplementary material for: Unraveling the Protective Role of Oleocanthal and Its Oxidation Product, Oleocanthalic Acid, against Neuroinflammation
Source: Antioxidants (Basel). 2024 Sep 3;13(9):1074. doi: 10.3390/antiox13091074 (PMC11428454; doi:10.3390/antiox13091074)
Supplement: Supplementary file 1 [file antioxidants-13-01074-s001.zip › antioxidants-3164811-supplementary.pdf]

Table S1. List of proteins found differentially expressed in the comparison of microglia cells treated with OL+LPS vs cells treated with LPS identified by LC-MS/MS. ID: SwissProt accession number, MW: molecular weight, pI: isoelectric point.									
**Spot**	**Accession**	**Gene name**	**Coverage (%)**	**#Peptides**	**#Unique**	**Avg. Mass**	**pI**	**ratio (OL+LPS/LPS)**	**Description**
304	Q8CGK3	LONM_MOUSE	3	2	2	105843	5.69	0.57	Lon protease homolog mitochondrial OS=Mus musculus OX=10090 GN=Lonp1 PE=1 SV=2
410	P13020	GELS_MOUSE	11	6	6	85942	5.72	2.67	Gelsolin OS=Mus musculus OX=10090 GN=Gsn PE=1 SV=3
419	Q99PL5	RRBP1_MOUSE	21	29	29	172878	9.35	1.86	Ribosome-binding protein 1 OS=Mus musculus OX=10090 GN=Rrbp1 PE=1 SV=2
425	P13020	GELS_MOUSE	33	18	10	85942	5.72	2.60	Gelsolin OS=Mus musculus OX=10090 GN=Gsn PE=1 SV=3
484	Q9JKF1	IQGA1_MOUSE	5	7	7	188741	6.07	0.38	Ras GTPase-activating-like protein IQGAP1 OS=Mus musculus OX=10090 GN=Iqgap1 PE=1 SV=2
486	P13020	GELS_MOUSE	14	9	4	85942	5.72	1.76	Gelsolin OS=Mus musculus OX=10090 GN=Gsn PE=1 SV=3
817	P61979	HNRPK_MOUSE	47	22	3	50976	5.39	1.71	Heterogeneous nuclear ribonucleoprotein K OS=Mus musculus OX=10090 GN=Hnrnpk PE=1 SV=1
919	Q61024	ASNS_MOUSE	27	13	13	64283	6.12	2.60	Asparagine synthetase [glutamine-hydrolyzing] OS=Mus musculus OX=10090 GN=Asns PE=1 SV=3
944	P27773	PDIA3_MOUSE	32	15	15	56678	5.69	0.58	Protein disulfide-isomerase A3 OS=Mus musculus OX=10090 GN=Pdia3 PE=1 SV=2
944	P63038	CH60_MOUSE	18	9	9	60956	5.39	0.58	60 kDa heat shock protein mitochondrial OS=Mus musculus OX=10090 GN=Hspd1 PE=1 SV=1
999	P30416	FKBP4_MOUSE	22	7	7	51572	5.54	1.90	Peptidyl-prolyl cis-trans isomerase FKBP4 OS=Mus musculus OX=10090 GN=Fkbp4 PE=1 SV=5
1009	Q8BGD9	IF4B_MOUSE	15	10	10	68840	5.46	1.65	Eukaryotic translation initiation factor 4B OS=Mus musculus OX=10090 GN=Eif4b PE=1 SV=1
1013	P63038	CH60_MOUSE	12	5	5	60956	5.35	0.41	60 kDa heat shock protein mitochondrial OS=Mus musculus OX=10090 GN=Hspd1 PE=1 SV=1
1014	P30416	FKBP4_MOUSE	34	15	15	51572	5.54	2.22	Peptidyl-prolyl cis-trans isomerase FKBP4 OS=Mus musculus OX=10090 GN=Fkbp4 PE=1 SV=5
1015	Q8JZK9	HMCS1_MOUSE	6	3	3	57569	5.65	1.79	Hydroxymethylglutaryl-CoA synthase cytoplasmic OS=Mus musculus OX=10090 GN=Hmgcs1 PE=1 SV=1
1027	Q61233	PLSL_MOUSE	12	6	6	70149	5.2	0.47	Plastin-2 OS=Mus musculus OX=10090 GN=Lcp1 PE=1 SV=4
1037	P62814	VATB2_MOUSE	21	9	9	56551	5.57	2.20	V-type proton ATPase subunit B brain isoform OS=Mus musculus OX=10090 GN=Atp6v1b2 PE=1 SV=1

1118	P16675	PPGB_MOUSE	11	5	5	53844	5.55	0.64	Lysosomal protective protein OS=Mus musculus OX=10090 GN=Ctsa PE=1 SV=1
1148	P54987	IRG1_MOUSE	24	11	11	53759	7.09	0.47	Cis-aconitate decarboxylase OS=Mus musculus OX=10090 GN=Acod1 PE=1 SV=2
1150	P54987	IRG1_MOUSE	38	16	16	53759	7.09	0.48	Cis-aconitate decarboxylase OS=Mus musculus OX=10090 GN=Acod1 PE=1 SV=2
1152	P26443	DHE3_MOUSE	42	20	20	61337	6.71	0.57	Glutamate dehydrogenase 1 mitochondrial OS=Mus musculus OX=10090 GN=Glud1 PE=1 SV=1
1164	Q8BWY3	ERF1_MOUSE	41	17	15	49031	5.51	1.56	Eukaryotic peptide chain release factor subunit 1 OS=Mus musculus OX=10090 GN=Etf1 PE=1 SV=4
1179	Q03265	ATPA_MOUSE	26	12	12	59753	8.28	0.45	ATP synthase subunit alpha mitochondrial OS=Mus musculus OX=10090 GN=Atp5f1a PE=1 SV=1
1180	Q03265	ATPA_MOUSE	27	13	13	59753	8.28	0.37	ATP synthase subunit alpha mitochondrial OS=Mus musculus OX=10090 GN=Atp5f1a PE=1 SV=1
1234	P54775	PRS6B_MOUSE	25	10	10	47408	5.09	0.59	26S proteasome regulatory subunit 6B OS=Mus musculus OX=10090 GN=Psmc4 PE=1 SV=2
1247	P99024	TBB5_MOUSE	15	6	6	49671	4.78	1.78	Tubulin beta-5 chain OS=Mus musculus OX=10090 GN=Tubb5 PE=1 SV=1
1337	P63038	CH60_MOUSE	13	9	9	60956	5.35	0.37	60 kDa heat shock protein mitochondrial OS=Mus musculus OX=10090 GN=Hspd1 PE=1 SV=1
1346	Q64345	IFIT3_MOUSE	46	17	17	47223	5.51	0.31	Interferon-induced protein with tetratricopeptide repeats 3 OS=Mus musculus OX=10090 GN=Ifit3 PE=1 SV=1
1380	P20152	VIME_MOUSE	40	19	5	53688	5.05	0.24	Vimentin OS=Mus musculus OX=10090 GN=Vim PE=1 SV=3
1535	Q61233	PLSL_MOUSE	19	10	10	70149	5.2	1.62	Plastin-2 OS=Mus musculus OX=10090 GN=Lcp1 PE=1 SV=4
1689	P07901	HS90A_MOUSE	10	7	7	84788	4.93	0.25	Heat shock protein HSP 90-alpha OS=Mus musculus OX=10090 GN=Hsp90aa1 PE=1 SV=4
1697	Q93092	TALDO_MOUSE	19	7	7	37387	6.57	1.61	Transaldolase OS=Mus musculus OX=10090 GN=Taldo1 PE=1 SV=2
1739	P45377	ALD2_MOUSE	8	3	3	36121	5.97	1.57	Aldose reductase-related protein 2 OS=Mus musculus OX=10090 GN=Akr1b8 PE=1 SV=2
1751	P14869	RLA0_MOUSE	19	4	4	34216	5.91	1.73	60S acidic ribosomal protein P0 OS=Mus musculus OX=10090 GN=Rplp0 PE=1 SV=3
1753	Q8CCF0	PRP31_MOUSE	15	6	6	55430	5.55	2.27	U4/U6 small nuclear ribonucleoprotein Prp31 OS=Mus musculus OX=10090 GN=Prpf31 PE=1 SV=3
1772	P14869	RLA0_MOUSE	50	18	18	34216	5.91	1.90	60S acidic ribosomal protein P0 OS=Mus musculus OX=10090 GN=Rplp0 PE=1 SV=3

1789	Q8CDN6	TXNL1_MOUSE	42	12	12	32237	4.84	0.49	Thioredoxin-like protein 1 OS=Mus musculus OX=10090 GN=Txnl1 PE=1 SV=3
1834	P11499	HS90B_MOUSE	11	8	8	83281	4.97	0.19	Heat shock protein HSP 90-beta OS=Mus musculus OX=10090 GN=Hsp90ab1 PE=1 SV=3
1867	O08585	CLCA_MOUSE	29	8	8	25604	4.5	4.43	Clathrin light chain A OS=Mus musculus OX=10090 GN=Clta PE=1 SV=2
1952	Q9R0P3	ESTD_MOUSE	17	3	3	31320	6.77	1.53	S-formylglutathione hydrolase OS=Mus musculus OX=10090 GN=Esd PE=1 SV=1
1982	Q9D8Y0	EFHD2_MOUSE	5	1	1	26791	5.01	0.51	EF-hand domain-containing protein D2 OS=Mus musculus OX=10090 GN=Efhd2 PE=1 SV=1
2037	P61290	PSME3_MOUSE	9	3	3	29506	5.69	3.74	Proteasome activator complex subunit 3 OS=Mus musculus OX=10090 GN=Psme3 PE=1 SV=1
2102	Q9R1P4	PSA1_MOUSE	38	11	11	29547	6	2.24	Proteasome subunit alpha type-1 OS=Mus musculus OX=10090 GN=Psma1 PE=1 SV=1
2158	P97372	PSME2_MOUSE	28	7	7	27057	5.55	0.57	Proteasome activator complex subunit 2 OS=Mus musculus OX=10090 GN=Psme2 PE=1 SV=4
2158	Q9CYZ2	TPD54_MOUSE	15	3	3	24043	5.8	0.57	Tumor protein D54 OS=Mus musculus OX=10090 GN=Tpd52l2 PE=1 SV=1
2252	P61982	1433G_MOUSE	32	9	6	28303	4.8	0.22	14-3-3 protein gamma OS=Mus musculus OX=10090 GN=Ywhag PE=1 SV=2
2279	P68254	1433T_MOUSE	6	1	1	27778	4.69	0.20	14-3-3 protein theta OS=Mus musculus OX=10090 GN=Ywhaq PE=1 SV=1
2288	Q9CQV8	1433B_MOUSE	27	6	6	28086	4.77	0.14	14-3-3 protein beta/alpha OS=Mus musculus OX=10090 GN=Ywhab PE=1 SV=3
2292	Q9JKB1	UCHL3_MOUSE	25	4	4	26152	4.96	0.26	Ubiquitin carboxyl-terminal hydrolase isozyme L3 OS=Mus musculus OX=10090 GN=Uchl3 PE=1 SV=2
2304	P62259	1433E_MOUSE	36	8	6	29174	4.63	0.34	14-3-3 protein epsilon OS=Mus musculus OX=10090 GN=Ywhae PE=1 SV=1
2363	Q61599	GDIR2_MOUSE	49	12	12	22851	4.95	1.60	Rho GDP-dissociation inhibitor 2 OS=Mus musculus OX=10090 GN=Arhgdib PE=1 SV=3
2556	Q9R1P3	PSB2_MOUSE	13	3	3	22906	6.52	0.65	Proteasome subunit beta type-2 OS=Mus musculus OX=10090 GN=Psmb2 PE=1 SV=1
2576	Q61171	PRDX2_MOUSE	62	12	12	21779	5.2	1.67	Peroxiredoxin-2 OS=Mus musculus OX=10090 GN=Prdx2 PE=1 SV=3
2653	Q9R0Q7	TEBP_MOUSE	13	2	2	18721	4.33	2.12	Prostaglandin E synthase 3 OS=Mus musculus OX=10090 GN=Ptges3 PE=1 SV=1
3359	P63323	RS12_MOUSE	29	4	4	14525	7.01	1.61	40S ribosomal protein S12 OS=Mus musculus OX=10090 GN=Rps12 PE=1 SV=2

3922	Q9D1Q6	ERP44_MOUSE	6	2	2	46853	5.08	0.37	Endoplasmic reticulum resident protein 44 OS=Mus musculus OX=10090 GN=Erp44 PE=1 SV=1
3929	Q9JHR7	IDE_MOUSE	7	6	6	117772	6.1	1.69	Insulin-degrading enzyme OS=Mus musculus OX=10090 GN=Ide PE=1 SV=1
3968	P08113	ENPL_MOUSE	16	12	12	92476	4.72	0.43	Endoplasmin OS=Mus musculus OX=10090 GN=Hsp90b1 PE=1 SV=2
4004	P20029	BIP_MOUSE	18	9	8	72422	5.01	1.51	Endoplasmic reticulum chaperone BiP OS=Mus musculus OX=10090 GN=Hspa5 PE=1 SV=3
4004	Q91YP2	NEUL_MOUSE	11	6	6	80429	5.7	1.51	Neurolysin mitochondrial OS=Mus musculus OX=10090 GN=Nln PE=1 SV=1
4030	Q8C1A5	THOP1_MOUSE	7	4	4	78026	5.72	0.52	Thimet oligopeptidase OS=Mus musculus OX=10090 GN=Thop1 PE=1 SV=1
4207	P18242	CATD_MOUSE	29	11	11	44954	5.63	2.12	Cathepsin D OS=Mus musculus OX=10090 GN=Ctsd PE=1 SV=1
4220	P13020	GELS_MOUSE	23	17	8	85942	5.72	0.44	Gelsolin OS=Mus musculus OX=10090 GN=Gsn PE=1 SV=3
4220	P55302	AMRP_MOUSE	29	8	8	42215	6.7	0.44	Alpha-2-macroglobulin receptor-associated protein OS=Mus musculus OX=10090 GN=Lrpap1 PE=1 SV=1
4345	Q9CQX2	CYB5B_MOUSE	8	1	1	16318	4.93	0.21	Cytochrome b5 type B OS=Mus musculus OX=10090 GN=Cyb5b PE=1 SV=1
4346	Q9CQX2	CYB5B_MOUSE	8	1	1	16318	4.79	0.31	Cytochrome b5 type B OS=Mus musculus OX=10090 GN=Cyb5b PE=1 SV=1
